# Domain analysis of the *Nematostella vectensis* SNAIL ortholog reveals unique nucleolar localization that depends on the zinc-finger domains

**DOI:** 10.1038/srep12147

**Published:** 2015-07-20

**Authors:** Ada A. Dattoli, Mark A. Hink, Timothy Q. DuBuc, Bram J. Teunisse, Joachim Goedhart, Eric Röttinger, Marten Postma

**Affiliations:** 1 Molecular Cytology, Swammerdam Institute for Life Sciences, University of Amsterdam Science Park 904, NL-1098 XH Amsterdam The Netherlands; 2 van Leeuwenhoek Centre for Advanced Microscopy (LCAM), University of Amsterdam, Science Park 904, NL-1098 XH Amsterdam The Netherlands; 3 The Whitney Laboratory for Marine Bioscience 9505 Ocean Shore Blvd, St. Augustine, FL 32080-8610; 4 Université Nice Sophia Antipolis, IRCAN, UMR 7284, 06107 Nice, France; 5 CNRS, IRCAN, UMR 7284, 06107 Nice, France; 6 INSERM, IRCAN, U1081, 06107 Nice, France

## Abstract

SNAIL transcriptional factors are key regulators during development and disease. They arose early during evolution, and in cnidarians such as *Nematostella vectensis*, *Nv*SNAILA/B are detected in invaginating tissues during gastrulation. The function of SNAIL proteins is well established in bilaterians but their roles in cnidarians remain unknown. The structure of *Nv*SNAILA and B is similar to the human SNAIL1 and 2, including SNAG and zinc-finger domains. Here, we performed a molecular analysis on localization and mobility of *Nv*SNAILA/B using mammalian cells and *Nematostella* embryos. *Nv*SNAILA/B display nuclear localization and mobility similar to *Hs*SNAIL1/2. Strikingly, *Nv*SNAILA is highly enriched in the nucleoli and shuttles between the nucleoli and the nucleoplasm. Truncation of the N-terminal SNAG domain, reported to contain Nuclear Localization Signals, markedly reduces nucleolar levels, without effecting nuclear localization or mobility. Truncation of the C-terminal zinc-fingers, involved in DNA binding in higher organisms, significantly affects subcellular localization and mobility. Specifically, the zinc-finger domains are required for nucleolar enrichment of *Nv*SNAILA. Differently from SNAIL transcriptional factors described before, *Nv*SNAILA is specifically enriched in the nucleoli co-localizing with nucleolar markers even after nucleolar disruption. Our findings implicate additional roles for SNAG and zinc-finger domains, suggesting a role for *Nv*SNAILA in the nucleolus.

The *Snail* gene family, first identified in flies^1^, encodes zinc-finger transcription factors probably best known for inducing changes in cell shape and morphology during cell migration^2^. Specifically, they are required for acquisition of cell motility through loss of adhesion in several organisms, including mammals^3^. The role of promoting cell migration is essential for the Epithelial- to- Mesenchymal Transition (EMT), a process that occurs during embryogenesis, where epithelial cells acquire fibroblast-like properties through down-regulation of epithelial genes and up-regulation of mesenchymal genes. *Snail* genes are directly involved in the transcriptional repression of *E-cadherin*, which is an important hallmark of EMT in many organisms^4^,^5^. This causes reduced intercellular adhesion and increased motility, such that cells are able to detach from the epithelial layer and become part of the mesenchyme^6^. It has also been shown that EMT is involved in other cellular processes including wound healing, chronic inflammation, fibrosis and malignant epithelial tumour progression^7^,^8^. Snail genes are also activated during other important cellular and developmental processes, such as dorsal- ventral pattering^9^, cell fate decision and right-left asymmetry^10^.

More than 150 *Snail* genes, including *Scratch* and *Slug*, have been identified in various taxa, such as vertebrates, non-vertebrate chordates, insects, nematodes, cnidarians and placozoans^3^,^11^. These transcription factors all share the same highly conserved C-terminal domain comprising at least four zinc fingers, either C_2_H_2_ motifs alone or C_2_H_2_ coupled with the C_2_HC variant. These zinc fingers can bind to the double-helix of DNA, recognizing specific motifs for regulation activity^12^. Apart from these four conserved zinc-fingers, Snail proteins can also contain one or two extra zinc-fingers. In contrast, the N-terminal domain is highly divergent and is thought to be involved in transcriptional regulation of E-cadherin expression in Bilateria via cofactor interaction^12^,^13^.

Snail family members are considered as important mesodermal determinants in triploblastic animals, which all possess three germ layers: ectoderm, endoderm and mesoderm. Surprisingly, they have also been discovered in diploblastic animals, such as cnidarians, which only possess an ectoderm and endoderm, and no true mesoderm. In these organisms, *snail* is predominantly expressed in the endoderm^14^,^15^. During cnidarian embryogenesis, Snail is expressed at the site of invagination in the presumptive endoderm prior to the onset of gastrulation. Comparatively, cnidarian Snail expression and the accompanying cellular movement is very similar to the one driving the formation of mesoderm in Bilateria during late gastrulation^3^,^16^.

Cnidarians are a sister group of the bilateria and therefore can provide insights into the evolution of gene families. They are a large and successful phylum of animals that diverged from the bilateria 600–800 million years ago^14^. Despite often being considered as simple or primitive, studies on different members of this phylum have revealed high molecular^17^ and morphological complexity^18^. *Nematostella vectensis*, like all cnidarians, lacks a true mesoderm layer, yet it exhibits a gene repertoire encoding many transcription factors involved in bilaterian development and mesoderm formation^14^,^19^. Intriguingly, so far no evidence has been found that cnidarians possess real EMT or have the ability to develop (metastatic) tumours. Although the role of snail during endomesoderm formation in higher metazoans is well established^20^, the only study reported to date did not find any obvious role for SNAILA and B during *Nematostella vectensis* gastrulation.

Specific domains important either for nuclear localization or for transcriptional activity have been well characterized for human, mouse*, Drosophila* and *Xenopus* Snail transcription factors, but very little is known about Snail family members from other species, making it challenging to compare these proteins within an evolutionary context.

## Results

### Sequence and domain analysis of *Nematostella vectensis* SNAIL

**Figure 1:**
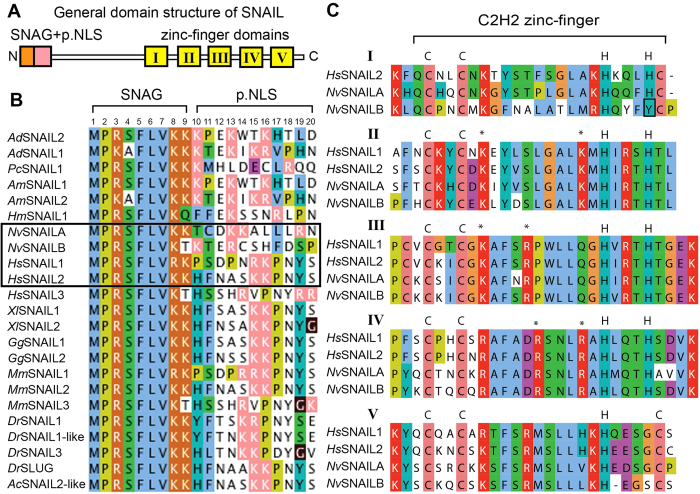
SNAIL domain analysis. **A**) General SNAIL structure. **B**) The amino acid residues shown in orange, included in the SNAG domain, are primarily involved transcriptional activation, through interactions with various cofactors, and also in nuclear localization. The subsequent amino acid sequence (10–20) contains many charged residues (pink) that may act as a Nuclear Localization Signal (NLS), which are less well conserved between higher and lower animals. The proteins shown in the rectangular box are included in this study. **C**) Alignment of the zinc-fingers of *Nv*SNAILA, B and *Hs*SNAIL1, 2 shows that *Nv*SNAILB first zinc-finger structure is not fully conserved. Colour codes shows the conservation of amino acids; the asterisk (*) indicates amino acids previously shown to be involved in nuclear localization^25^.

**Figure 2:**
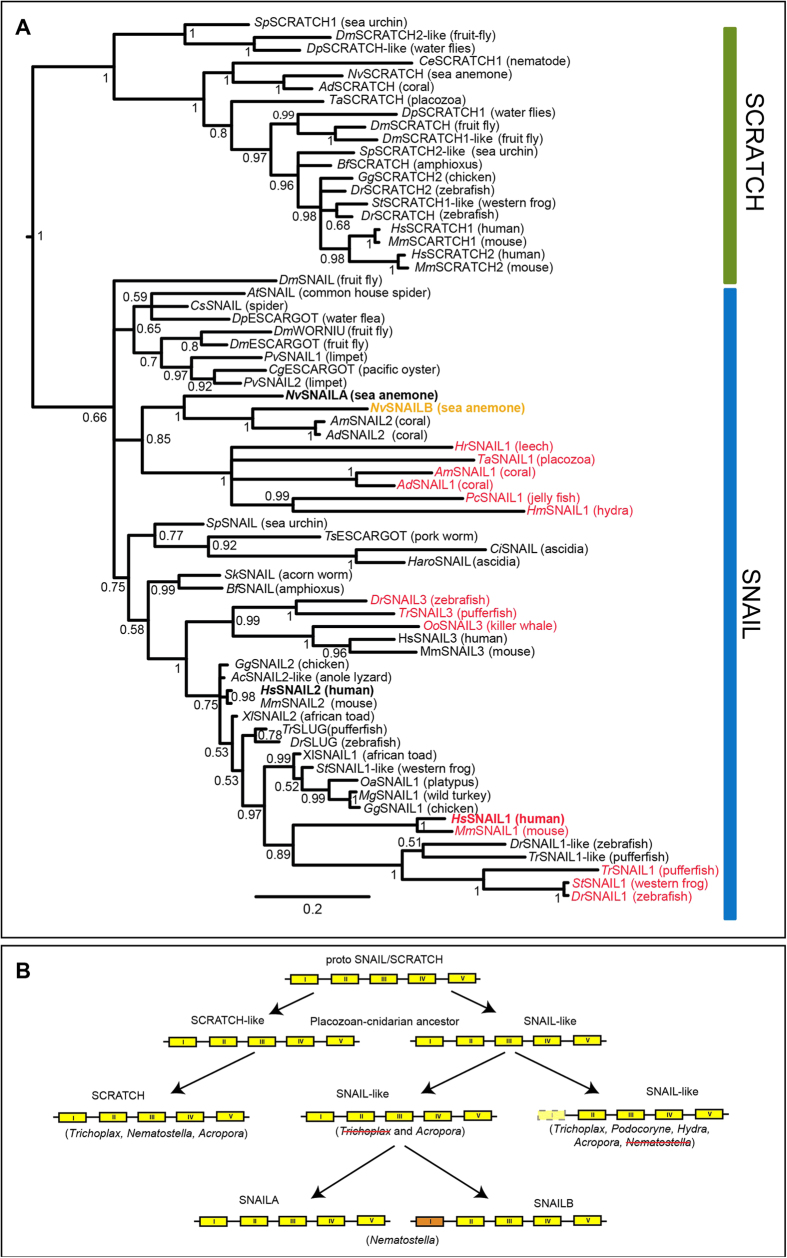
**A**) Phylogenetic tree of related Scratch and Snail zinc-finger proteins. This tree was based on the alignment of the sequence region that comprised the five zinc-finger domains (see Methods). C2H2 motif prediction was performed on all sequences using SMART and the proteins were colour coded based on the predicted first zinc-finger domain. Proteins in black are predicted to have the first zinc-finger domain, comprising a total of five zinc-finger domains. Proteins in red are predicted to have only four zinc-finger domains, hence missing the first zinc-finger domain. The first zinc-finger of *Nv*SNAILB (orange) is weakly recognized (E-value 0.25; see supplementary dataset 4 for SMART predictions). This analysis shows that the number of zinc fingers among snail proteins is variable across species and indicates multiple duplication events, zinc-finger domain loss and gene loss. *Phylogenetic analysis*. C2H2 zinc-finger domains where predicted using the Simple Modular Architecture Research; Multiple sequence alignment was performed with Muscle 3.8.31 software^58^ and were subsequently manually improved (in MacVector 11.0.2). Handling of the multiple sequence alignment was done using jalview 2.7 software^59^. Tree reconstruction was performed with MR.BAYES3^60^, the consensus tree shown was based on 2,000,000 generations. Trichoplax adhaerens (*Ta*) SNAIL (XP_002108251) has been raced out from Trichoplax adhaerens coding DNA for this study (see primer sequences in supplementary information). **B**) Proposed evolutionary history of Snail. A proto SNAIL/SCRATCH gene was duplicated early during evolution (Placozoan-cnidarian-bilaterian) ancestor, giving rise to two genes, of which one evolved into the Scratch family and the other into the Snail family. A subsequent duplication event gave rise to two *snail* genes, of which one lost the first zinc-finger domain and the second retained the first zinc-finger domain. *Nematostella vectensis* comprises two *snail* genes, SNAILA and SNAILB, of which the latter still has a partial first zinc-finger domain but was not completely lost. This can indicate that *Nematostella vectensis* lost the gene with only four zinc-finger domains and underwent another duplication event with the five zinc-finger domains SNAIL or that the first zinc-finger domain of *Nv*SNAILB more slowly evolved compared to other species.

To be able to characterize the cellular behaviour of SNAILA and, B, we first made a detailed comparison of structures and sequences of snail orthologs from the animal kingdom. Snail proteins belong to the Snail superfamily, which includes also Scratch and Slug proteins. They usually are (with few exceptions) about 270 amino acids long; their N-terminus (generally the first 20 amino acids) exhibits high variability across species. It usually comprises the N-terminal SNAG domain (Fig. 1A,B), represented by a short sequence of nine amino acids (amino acids 1–9) and also several positively charged residues reported to be involved in nuclear localisation^21^,^22^. The SNAG domain has been reported to be involved in interactions with cofactors, specifically the arginine in 3^rd^ position and the serine in 4^th^ seem to be specifically involved in transcriptional activity through interaction with cofactors whereas arginine and lysine at the 8^th^ and 9^th^ position have been reported also to act as a nuclear localisation signal (NLS)^12^,^23^,^22^,^24^. The alignment shown in Fig. 1 (Fig. 1B) shows the high conservation in the SNAG domain between cnidarians and vertebrates. Both *Nv*SNAILA and *Nv*SNAILB comprise a conserved SNAG domain and also several positively charged residues at the N-terminal. Furthermore, all proteins consist of one or more short sequences of positively charged lysine (K) or arginine (R), which may act as a putative NLS. For vertebrates such as humans, mice and amphibians some of these overlap with the SNAG domain (R3, K/R8K9 in orange) and others are located in position 15 and 16 of the N-terminal of snail proteins (KK or RK shown in pink)^21^,^22^. Also the cnidarian representatives still have the same putative NLS sequence but they are found at different positions, for instance K13–14 for *Nv*SNAILA and K10- R13 for *Nv*SNAILB instead of being located at residue 15–16. Due to the high variability of the N-terminal domain, it is often excluded from phylogenetic analysis^11^, and rather the C-terminal of the protein comprising the zinc-finger domains (generally starting from amino acid 120) is more suitable. Snail family members all possess at least four zinc-finger domains (Znf II-V), and often a fifth zinc-finger domain (Znf I) is found N-terminally to Znf II. The four zinc-finger domains at the C-terminus are reported as essential for DNA binding and also comprise a nuclear localisation signal^22^,^25^. In order to carefully group snail family proteins, we constructed a tree based on 69 snail super-family proteins comprising 49 snail and 20 scratch proteins (Fig. 2A) using the alignment of the region that covers the five zinc–finger domains (see supplementary dataset 2,3 for accession numbers and sequence alignment). We used the Simple Modular Architecture Research Tool (SMART,^26^) to predict the presence of zinc fingers in each protein (see supplementary information for SMART E-values); the scratch proteins were used as an out-group. From the data obtained, cnidarians are the first animals to have duplicated snail isoforms and interestingly, the sea anemone *Nematostella vectensis* has two snail proteins previously stated as having both five zinc-finger domains: SNAILA and SNAILB (Znf I-V)^11^,^27^. Finally, vertebrates have at least three SNAIL representatives. In this phylum, among the snail proteins, fish, mice, frogs and humans have one snail representative containing four zinc-finger domains (Znf II-V). Similarly to what has been previously shown^11^, these results show that animals usually have (at minimum) a single snail gene with four zinc-finger domains and a second gene with five zinc-finger domains. It is likely that the loss of the first zinc-finger domain occurred in several lineages over time. Both *Nv*SNAILA and B have been previously described as SNAIL proteins containing five zinc fingers. However, since loss of the first zinc seems to be occurred several times across the evolution we decided to look more carefully at the sequences of *Nv*SNAILA, B compared to vertebrates SNAILs respectively possessing 4 and 5 zinc finger. As shown in the alignment (Fig. 1C) the first zinc-finger domain of *Nv*SNAILB is different compared to *Nv*SNAILA and *Hs*SNAIL 1 and 2 used as vertebrate representatives with 4 and 5 zinc fingers, respectively and appears to be partial as the second histidine is lacking. Together these results indicate that *Nv*SNAILA and B show high conservation of the SNAG, putative NLS and zinc finger domains compared to vertebrates, however despite being often characterized as a five zinc-finger domains SNAIL, *Nv*SNAILB might actually have lost the first finger.

### Cellular localization of *Nv*SNAILA, *Nv*SNAILB, *Hs*SNAIL1 and *Hs*SNAIL2 in HeLa cells

**Figure 3:**
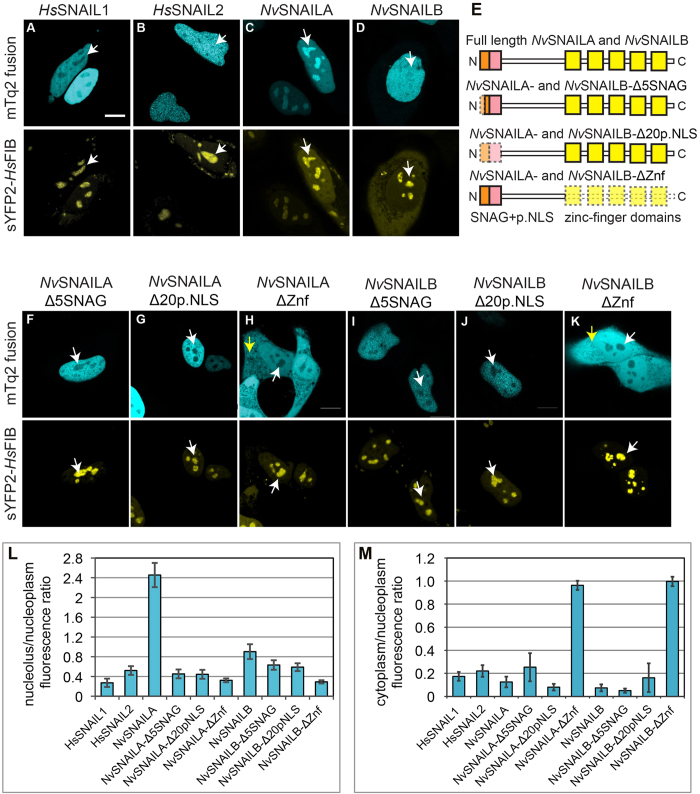
Cellular localisation of *Nv*SNAILA and B constructs and their truncated variants in HeLa cells. **A**–**D**) Cellular localisation by confocal imaging of the full length (FL) constructs of *Nv*SNAILA, B and *Hs*SNAIL1, 2 fused to mTurquoise2 at the C-terminus in HeLa cells together with the nucleolar marker sYFP2-*Hs*FIB, white arrows indicate position of nucleoli. **E**) Constructs analysed for both *Nv*SNAILA and *Nv*SNAILB by truncation of respectively the first 5 amino acids of the SNAG domain alone (*Nv*SNAILA/B-Δ5SNAG), SNAG domain together with the predicted NLS (*Nv*SNAILA/B-Δ20p.NLS) and of the entire zinc-finger domain (*Nv*SNAILA/B-ΔZnf). **F**–**K**) Confocal imaging of the truncated proteins transfected in HeLa cells fused to mTurquoise2 at the C-terminus together with the nucleolar marker sYFP2-*Hs*FIB, white arrows indicate position of nucleoli and yellow arrows the cytoplasm. Scale bar 10 μm. **L**) Quantification of nucleolar localisation expressed as the ratio of the fluorescence intensity in the nucleolus over nucleoplasm. Ratios are corrected for non-specific localisation using mCherry channel, zero values indicate non-specific localisation. Error bars are confidence intervals at 95% confidence level. **M**) Cytoplasm/nucleoplasm fluorescence ratios for different SNAIL constructs. All ratios are normalised with the ratio of mCherry, in this way any variation coming from geometrical differences or organelle distribution are removed from the ratio. If the ratio is equal to one, then the cytoplasm/nucleoplasm ratio is indistinguishable from mCherry. Error bars represent 95% confidence intervals.

**Figure 4:**
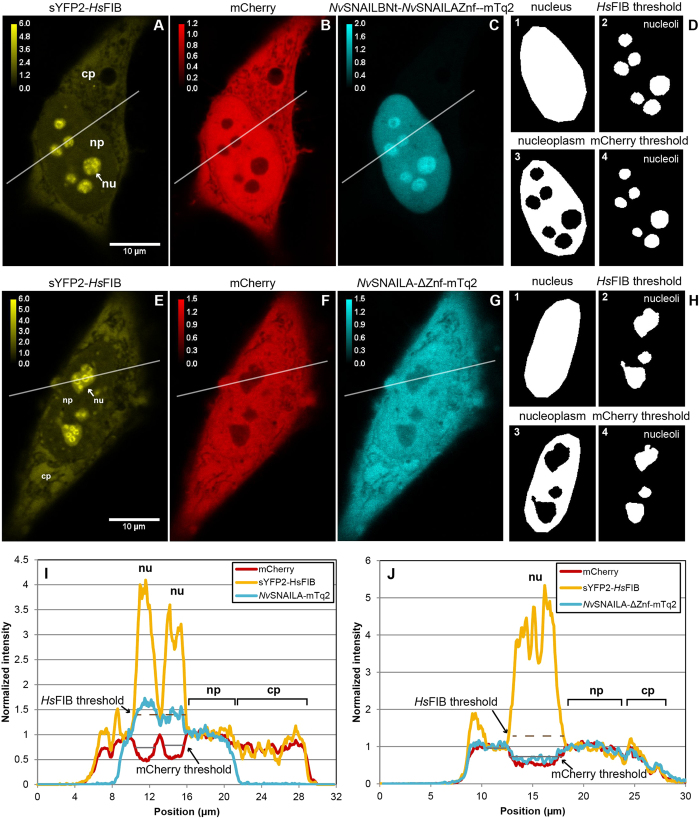
The quantification of localization is based on three confocal microscopy images with different colours. Based on three confocal microscopy images of the nucleolar marker *Hs*FIB (**A,E** fused to sYFP2), free mCherry (**B,F**) and the construct of interest (**C,G** fused to mTurquoise2) transfected in HeLa cells we extracted the cytoplasm/nucleoplasm ratio and nucleolus/nucleoplasm ratio. A background ROI was manually drawn in a region that was void of cells in all three channels, and each channel was background subtracted using the mean background fluorescence calculated from the background ROI. Subsequently a region of interest was manually drawn in the cytoplasm, and the median fluorescence value was calculated for all channels. Another region of interest was manually drawn in the nucleus, such that it encompassed the nucleoli that were visible in the *Hs*FIB image (**D1,H1**); care was taken not to include any cytoplasm. The mode in the nuclear ROI was calculated for both *Hs*FIB and mCherry and used to normalize the fluorescence of both channels and the initial estimate of the nucleolar mask was obtained by thresholding the mode normalized ratio image of *Hs*FIB/mCherry (at ratio level 2). An initial nucleoplasm mask was obtained by subtracting the initial nucleolar mask from the nucleus mask. Based on the pixels in the initial nucleoplasm mask a mean spatial profile (column profile, vertical direction of confocal scanning) was calculated and smoothed using boxcar averaging (11 pixels). The nucleus fluorescence (both nucleoplasm and nucleoli) for all the channels was subsequently normalized with their respective profiles, such that the nucleoplasm fluorescence was normalized to unity. The final nucleolar mask was then obtained by thresholding the normalized *Hs*FIB image using a value of 1.4 (**D2,H2**). The final nucleoplasm mask was obtained by subtracting the final nucleolar mask from the nucleus mask (**D3,H3**). Nucleoli were manually selected from the nucleolar mask and for each selected nucleolus the full width half minimum was determined from a smoothed normalized mCherry image (7 × 7 pixels Gaussian filter with a sigma of 200 nm). A refined nucleolar mask was then obtained by thresholding each nucleolar region using the full width half minimum of each nucleolar region (**D4**,**H4**). **I,J**) Line scan profiles of the normalized fluorescence level in each image (**A**–**C** and **E**–**G**).

In order to study the cellular localization of *Nematostella vectenis* snail proteins in comparison with human homologues, we transfected plasmids encoding *Nv*SNAILA, *Nv*SNAILB, *Hs*SNAIL1 and *Hs*SNAIL2 fused to mTurquoise2^28^ at the C-terminus in HeLa cells. This cell type was used as a heterologous model system for our comparison studies because many experimental cycles can be performed easily (transfection and advanced microscopy), constituting a convenient and powerful tool for protein analysis to combine with *Nematostella vectenis* embryological experiments. Previous studies have shown that the concentration levels of transcription factors in animal cells are found in a range of 10–300 nM^29^. These values are comparable with the values found with Fluorescence Correlation Spectroscopy (FCS) experiments (see Methods); values are about 5 fold higher for experiments performed with confocal microscopy. All over-expressed full-length SNAIL constructs exhibit strong nuclear localization compared to non-specific localisation of mCherry. The cytoplasm/nucleoplasm ratio of mCherry is *R*_*cp−np*_ = 0.89 ± 0.01 (*n* = 275) and relative to this ratio the full length constructs are about five-fold higher in the nucleoplasm (Fig. 3M and supplemental Table S2, see Fig. 4B,F for typical mCherry localisation). Furthermore, *Hs*SNAIL1 and 2 (Fig. 3A,B) show the same nuclear localization as previously described^21^,^25^. Because the protein levels in the nucleolus varied for the different SNAIL constructs (Fig. 3A–D) we quantified the ratios of nucleolus (*nu*) and nucleoplasm (*np*) (supplemental Table S2, Fig. 3L). With the aid of the nucleolar marker *Hs*FIB fused to the sYFP2^30^ and mCherry alone we extracted the nucleolus/nucleoplasm ratios of the constructs of interest and corrected for non-specific localisation to increase the sensitivity of the quantitative analysis (Fig. 4, see Methods). The quantitative analysis reveals that *Nv*SNAILA (Fig. 3A) is significantly enriched in the nucleoli (Fig. 3L), *R*_*nu−np*_ = 2.46 ± 0.25 (*n* = 27). This was also observed in other human cell lines such as HEK293 and U2OS (data not shown). We found that the N-terminal fusion construct, mTurquoise2-*Nv*SNAILA exhibited a similar enrichment level in nucleoli (data not shown) suggesting that the fused fluorescent protein is not interfering with the sub-cellular localisation mechanism. *Hs*SNAIL1 (Fig. 3A) exhibits significantly lower levels *R*_*nu−np*_ = 0.27 ± 0.09 (*n* = 31). *Nv*SNAILB (Fig. 3D) and *Hs*SNAIL2 (Fig. 3B) exhibit intermediate levels with ratios *R*_*nu−np*_ = 0.90 ± 0.15 (*n* = 14) and *R*_*nu−np*_ = 0.52 ± 0.09 (*n* = 32) respectively. Summarizing, *Nv*SNAILA displays strong nucleolar localization compared to the other constructs, while *Nv*SNAILB as well as *Hs*SNAIL1 and 2 are detected at much lower levels in this sub-nuclear structure and in fact are relatively excluded.

### *Nv*SNAILA specifically localizes at the Granular Component of HeLa cells nucleoli

**Figure 5:**
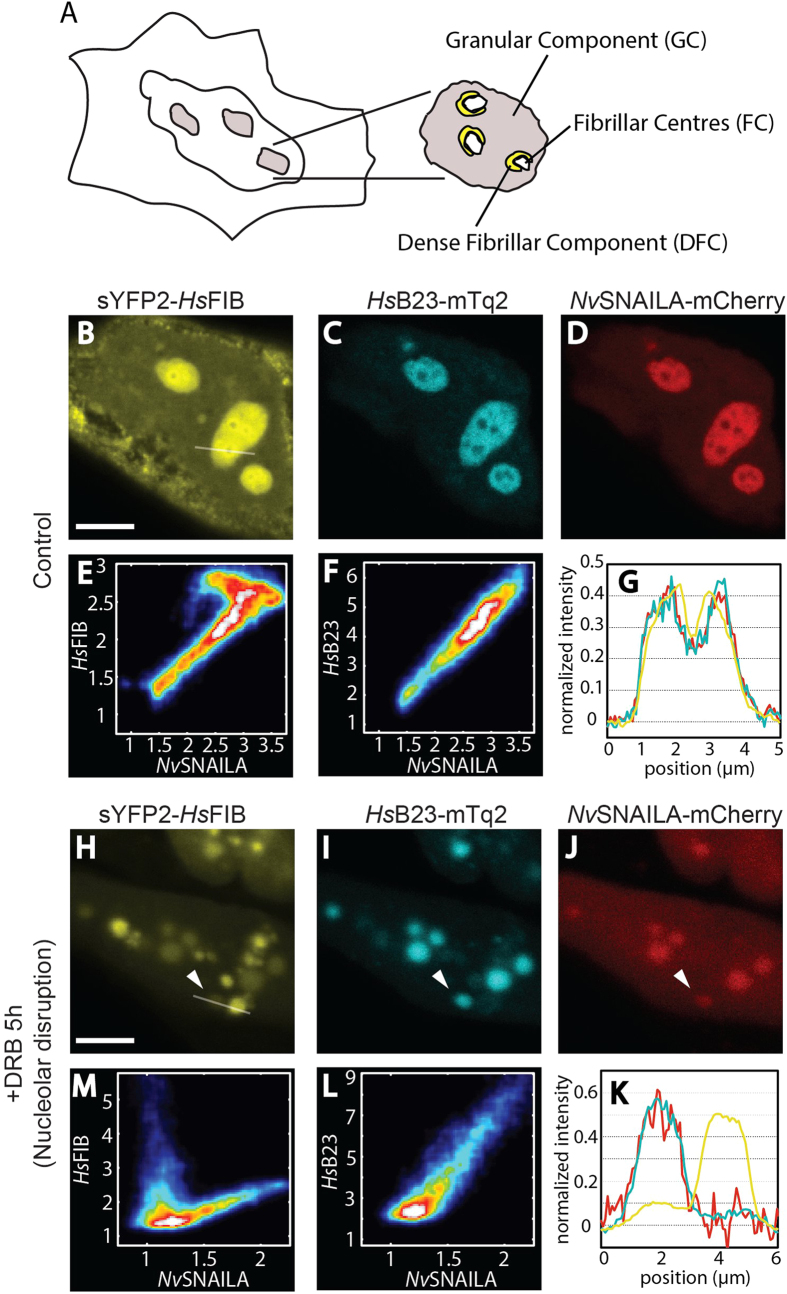
Localisation analysis of *Nv*SNAILA in combination with several nucleolar markers after DRB-induced nucleolar disruption. **A**) Diagram of the nucleolar structure. **B**–**D**) Control HeLa cells showing the specific nucleolar localization of *Hs*FIB, *Hs*B23 and *Nv*SNAILA. **E,F**) Smoothed scatter-plot of pixel intensities in the nucleoli for the pair *Nv*SNAILA-*Hs*FIB and *Nv*SNAILA-*Hs*B23. The pixel intensities in the nucleoli were normalized with respect to the nucleoplasm intensity. **G**). Fluorescence intensity profile of the three proteins across a region containing a nucleolus (white line in **B**). **H**–**J**) Separation of the DFC from the GC due to 5 h DRB treatment. **M-L**) Smoothed scatter-plot of *Nv*SNAILA-*Hs*FIB and *Nv*SNAILA-*Hs*B23. Only pixels that have higher intensity than the nucleoplasm were used, **K**). Plot of the fluorescence intensity profile of the three proteins in a region containing reminiscent nucleolar proteins (white line in **H**). Scale bar 10 μm.

In nucleoli from higher eukaryotes three different compartments or regions have been identified by electron microscopy^31^: Fibrillar Centres (FCs) containing the ribosomal DNA for 18S, 5.8S and 28S rRNA, Dense Fibrillar Component (DFC) and the Granular Component (GC) that surrounds FC and DFC (Fig. 5A). In an active nucleolus, transcription of the ribosomal genes occurs at the interface between FC and DFC, where also important modifications of the rRNAs take place^32^. The GC is associated with assembly of the complex ribosome machinery at late steps of rRNA processing^33^.

In order to determine the specific nucleolar regions at which *Nv*SNAILA localizes, we analysed the co-expression of *Nv*SNAILA with the human nucleolar markers *Hs*FIB, *Hs*NCL, *Hs*B23 and *Hs*SENP3 (Fig. 5 and supplemental Fig. S1). When co-expressing *Hs*FIB-sYFP2, *Hs*B23-mTq2 and *Nv*SNAILA-mCherry we observed that *Hs*FIB, a DFC marker, is highly enriched in the DFC, but also present in the GC and not in the FC (Fig. 5B). For *Hs*B23, a marker for the GC^34^, we observed that it is indeed more localized at the GC and does not show enrichment at the DFC nor the FC (Fig. 5C). *Nv*SNAILA exhibits a highly similar distribution compared to *Hs*B23, and is predominantly localized at the GC and not the DFC nor the FC (Fig. 5D). To further quantify this observation we plotted the pixel intensity obtained from the nucleolar regions for the pair *Hs*FIB-*Nv*SNAILA and for the pair *Hs*B23-*Nv*SNAILA. The scatter plot obtained for *Hs*FIB-*Nv*SNAILA shows that this pair does not show a pure linear relationship meaning that they do not co-localize in every region of the nucleolus (Fig. 5E), while for the couple *Hs*B23-*Nv*SNAILA a linear correspondence was found (Fig. 5F), meaning that they highly co-localize. This observation is further corroborated by comparing the normalized intensity profiles obtained for the three proteins in individual nucleoli (Fig. 5G); the intensity profile for the cell in Fig. 5B–D shows that *Hs*B23 and *Nv*SNAILA show a high degree of overlap, in contrast to *Hs*FIB, which exhibits higher intensities in the region with lower *Nv*SNAILA and *Hs*B23 intensities. Similar patterns were found for all the analysed cells (*n* = 20 cells). A similar result was found when *Nv*SNAILA was co-expressed with *Hs*SENP3 (*n* = 10 cells), another nucleolar marker for the GC^34^ (supplemental Fig. S1A–D). We finally investigated the co-localization between *Nv*SNAILA and *Hs*NCL, a nucleolar marker for both the GC and the DFC. In this case (*n* = 10 cells) we found that co-localization was similar to *Nv*SNAILA-*Hs*FIB (Fig. S1I–L) These results suggest that *Nv*SNAILA localizes at the Granular Component of nucleoli in HeLa cells together with the GC markers *Hs*B23 and *Hs*SENP3 and does not exhibit pronounced enrichment at the DFC or FC.

### *Nv*SNAILA co-localizes with *Hs*B23 and SENP3 after DRB-induced nucleolar disruption

To further investigate the sub-nucleolar localisation of *Nv*SNAILA and human nucleolar markers we induced nucleolar disruption using DRB, a casein kinase II (CK2) inhibitor that causes disconnection between the DFC and the GC^35^. In cells co-expressing *Hs*FIB, *Hs*B23 and *Nv*SNAILA incubated for 5 h with DRB we clearly observed disruption of the nucleoli (Fig. 5H–J). The nucleolus loses its organized architecture and the nucleolar marker proteins all exhibit a less well defined localization within the nucleus. In DRB treated cells (*n* = 15) we observed that *Nv*SNAILA becomes notably more uniformly distributed throughout the nucleoplasm compared to *Hs*B23 and *Hs*FIB, but 73% of the cells still showed residual enriched structures that strongly co-localize with *Hs*B23. *Hs*FIB exhibits two pools, one weakly co-localising and one pool that was not co-localising with *Hs*B23 and *Nv*SNAILA, the former is associated with remnants of the GC and the latter with remnants of the DFC (Fig. 5H–J, white arrows). This observation was also confirmed by both the scatter-plots (Fig. 5M-L) and the intensity profile (Fig. 5K). In the first case, the relation *Nv*SNAILA-*Hs*FIB clearly appears to be non-linear compared to the relation *Nv*SNAILA-*Hs*B23 as well as the intensity profiles show a high degree of co-localisation between *Nv*SNAILA and *Hs*B23 compared to *Hs*FIB. In cells co-transfected with the other nucleolar markers, *Hs*NCL and *Hs*SENP3, we observed similar effects on *Nv*SNAILA distribution and co-localisation (supplemental Fig. S1). *Nv*SNAILA exhibited enriched residual regions in 60% of the cells (*n* = 10), which co-localised with *Hs*SENP3 (Fig. S1E-F). Cells co-expressing NvSNAILA and *Hs*NCL exhibited two pools similar to *Hs*FIB, with a similar co-localisation pattern (Fig. S1M-N). All observations were further confirmed by the calculation of scatter-plots and intensity profiles (Fig. S1G-H and S1O-P). Nucleolar disruption by DRB was completely reversed within 20–60 min after washing away DRB, restoring the co-localisation patterns observed in untreated cells (data not shown). These observations indicate that *Nv*SNAILA co-localises with both *Hs*B23 and *Hs*SENP3 in the GC.

### Putative NLS does not affect nuclear localization of both *Nv*SNAILA and B while the SNAG domain contributes to their nucleolar localization

In order to investigate whether the N-terminal domain of *Nv*SNAILA,B is involved in sub-cellular/sub-nuclear localisation, two truncated versions fused to mTurquoise2 were analysed (Fig. 3E). The first truncation comprised the first five amino acids (Δ5SNAG), thereby removing the R in the third position of the SNAG domain. The second truncation comprised the first twenty amino acids (Δ20pNLS), thereby removing the complete sequence of the putative NLS and SNAG domain. The constructs were transfected in HeLa cells and their cellular localisation was analysed by confocal microscopy (Fig. 3F–I). Both Δ5SNAG and Δ20pNLS versions of *Nv*SNAILA and *Nv*SNAILB are still localized in the nucleus similar to the full-length proteins (Fig. 3C,D). However, the truncated versions of *Nv*SNAILA exhibit reduced levels in nucleoli (Fig. 5L, supplemental Table S2), with *Nv*SNAILA-Δ5SNAG, *R*_*nu−np*_ = 0.45 ± 0.09 (*n* = 12) and *Nv*SNAILA-Δ20pNLS, *R*_*nu−np*_ = 0.44 ± 0.09 (*n* = 14) showing a pronounced reduction compared to the full-length. Interestingly, the nucleolar localisation of *Nv*SNAILB-Δ5SNAG, *R*_*nu−np*_ = 0.63 ± 0.10 (*n* = 14) and *Nv*SNAILB-Δ20pNLS, *R*_*nu−np*_ = 0.59 ± 0.08 (*n* = 14) was also slightly but significantly reduced compared to the full-length protein. These results indicate that the first 5 aminoacids of the N-terminal domain are involved in nucleolar enrichment of *Nv*SNAILA and NvSNAILB.

### Truncation of the five zinc-finger domains of both *Nv*SNAILA and *Nv*SNAILB increases cytoplasmic levels and reduces nucleolar levels

As previously mentioned, both *Nv*SNAILA and *Nv*SNAILB contain four highly conserved zinc-finger domains (Znf II-V, Fig. 1C) and one zinc-finger (Znf I) which is less well conserved. For other species it has been reported that the last four zinc-finger domains are directly involved in DNA binding and also comprise residues that are involved in nuclear localisation^22^,^25^. In order to investigate if these domains have similar localization properties in *Nematostella vectensis* SNAILA and B, all C-terminal amino acids starting at position 123 were truncated, thereby removing the five zinc-finger domains (Fig. 3E), and the remaining protein was fused to mTurquoise2. Images were taken using confocal microscopy. Compared to their full-length versions, both *Nv*SNAILA-ΔZnf and *Nv*SNAILB-ΔZnf exhibit a marked increase in cytoplasmic levels (Fig. 3J,K,M, supplemental Table S2). Apart from losing specific nuclear localisation, both constructs also show a marked reduction in nucleolar levels with ratios of *Nv*SNAILA-ΔZnf, *R*_*nu−np*_ = 0.32 ± 0.04 (*n* = 13) and *Nv*SNAILB-ΔZnf, *R*_*nu−np*_ = 0.29 ± 0.03 (*n* = 16), similar to mCherry alone (Fig. 3L). Truncation of only the last four zinc-finger domains in *Nv*SNAIL (*Nv*SNAILA-ΔZnfII-V) resulted in a cellular distribution similar to *Nv*SNAILA-ΔZnf, (data not shown). Thus, we observed that both the five Zn-finger domains, and the SNAG domain, play crucial roles in nucleolar enrichment of *Nv*SNAILA.

### Cellular localization of *Nv*SNAILA and *Nv*SNAILB in *Nematostella vectensis* embryos

**Figure 6:**
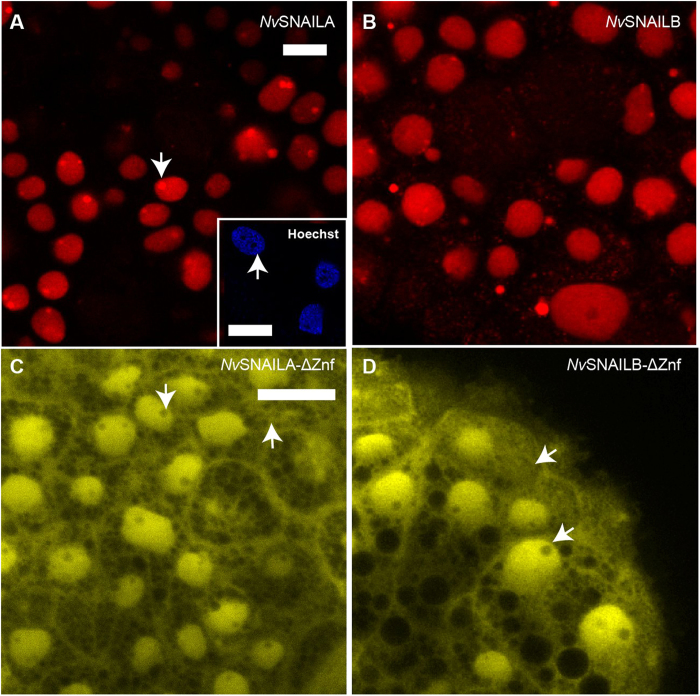
Cellular localisation of *Nv*SNAILA and B constructs in *Nematostella vectensis* embryos. **A**) Wild-type *Nv*SNAILA fused to mCherry specifically localizes into the nucleus and exhibits enrichment in nuclear regions that have similar size and shape as regions that are excluded for Hoechst (inset), a DNA marker that is not present in nucleoli (white arrows). **B**) Wild-type *Nv*SNAILB fused to mCherry specifically localizes into the nucleus exhibiting reduced levels in nucleoli-like regions compared to *Nv*SNAILA (white arrows). **C**,**D**) Mutant version of *Nv*SNAILA and B fused to mVenus lacking all the zinc-finger domains no longer specifically localize into the nucleus and is increased in the cytoplasm but is strongly reduced in nuclear regions similar to nucleoli.

To determine whether the cellular localisation observed in HeLa cells of *Nv*SNAIL mutants is conserved in *Nematostella vectensis* embryos, eggs were injected prior to fertilization with the RNA of each construct *Nv*SNAILA and, B and their respective truncated versions *Nv*SNAILA,B-ΔZnf. Once fluorescence was apparent in the fertilized embryos live images were taken. As shown in Fig. 6, the full length *Nv*SNAILA fused to the fluorescent protein mCherry specifically localizes into the nucleus and exhibits enrichment in regions with a similar size and shape that are excluded in embryos stained with Hoechst, a DNA marker that is excluded from nucleoli^36^ (Fig. 6A, inset). *Nv*SNAILB fused to the fluorescent protein mCherry also specifically localizes to the nucleus, but with reduced levels in nucleoli-like regions compared to *Nv*SNAILA (Fig. 6B). The cellular distribution of *Nv*SNAILA-ΔZnf , *Nv*SNAILB-ΔZnf fused to mVenus was also similar as observed in HeLa cells, exhibiting a marked increase in cytoplasmic levels as well as a marked reduction in nucleoli (Fig. 6C,D). We also injected *Nv*SNAILA-Δ5SNAG and Δ20pNLS mutants fused to mVenus, which also specifically localized into the nucleus and in a subset of nuclei exhibited reduced but more variable fluorescence distribution with respect to the nucleoli (data not shown).

### The zinc-finger domain region of *Nv*SNAILA is required for nucleolar enrichment

**Figure 7:**
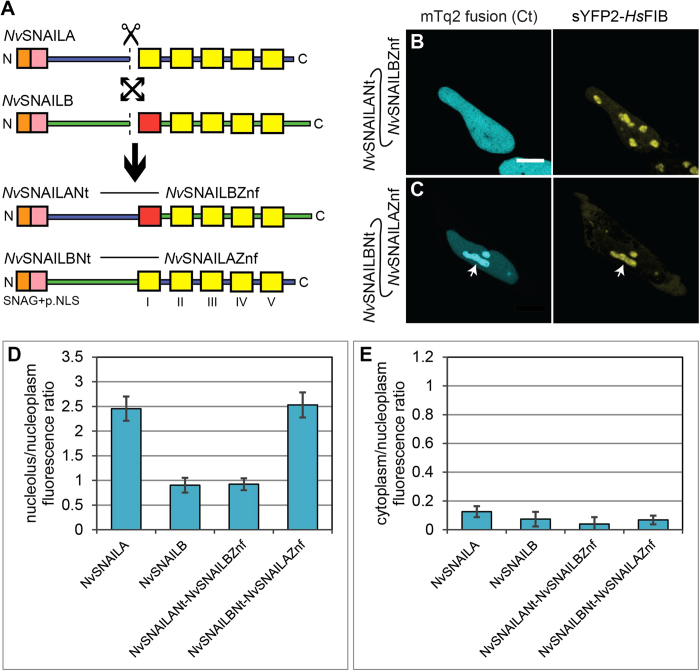
Localisation analysis of *Nv*SNAILA and *Nv*SNAILB hybrid constructs. **A**) The N-terminal domain and zinc-finger domains of *Nv*SNAILA and *Nv*SNAILB were swapped, thereby creating two hybrid versions. **B,C**) Hybrid constructs were transfected in HeLa cells and imaged by confocal microscopy, white arrows indicate position of nucleoli. **D**,**E**) Quantification of nucleolar and cytoplasmic localisation. Error bars are confidence intervals at 95% confidence level, scale bar 10 μm.

As shown in Fig. 3, *Nv*SNAILB exhibits moderate nucleolar levels compared to *Nv*SNAILA, while having the same first five N-terminal amino acids (Fig. 1B) and truncation of the first five and twenty amino acids of the SNAG domain as well as the zinc-finger domains results in marked nucleolar reduction of both *Nv*SNAILA and B in HeLa cells (Fig. 3). The last four zinc-finger domains are highly conserved between the different SNAIL proteins but the region between the SNAG domain and the first zinc-finger domains exhibits high sequence variability. Furthermore, the first zinc-finger domain of *Nv*SNAILB is different compared to *Nv*SNAILA, and appears to be partial as the second histidine is lacking (Fig. 1C). In order to further investigate the role of the N-terminal (SNAG up to first zinc-finger) and the C-terminal domain (all five zinc-finger domains) *Nv*SNAILA/*Nv*SNAILB hybrids were created. In one case, the N-terminal of *Nv*SNAILA and the zinc-finger domains of *Nv*SNAILB were combined (*Nv*SNAILANt-*Nv*SNAILBZnf) and vice versa, the N-terminal of *Nv*SNAILB and the zinc-finger domains of *Nv*SNAILA were combined (*Nv*SNAILBNt-*Nv*SNAILAZnf) (Fig. 7A). Both were fused to the fluorescent protein mTurquoise2 and their localisation was studied by confocal microscopy in HeLa cells. The localization of the hybrid constructs was compared with their wild type versions. As shown in Fig. 7B–D the hybrid *Nv*SNAILANt-*Nv*SNAILBZnf containing the zinc-finger domains of *Nv*SNAILB exhibits a similar distribution as full length *Nv*SNAILB. The hybrid *Nv*SNAILBNt-*Nv*SNAILAZnf with the zinc-finger domains of *Nv*SNAILA exhibits a similar distribution as full length *Nv*SNAILA, clearly showing that the zinc-finger domain region of *Nv*SNAILA contains information that drives nucleolar enrichment of the hybrid protein.

### Mobility of *Nv*SNAILA, *Nv*SNAILB, *Hs*SNAIL1 and *Hs*SNAIL2 in sub-cellular compartments

**Figure 8:**
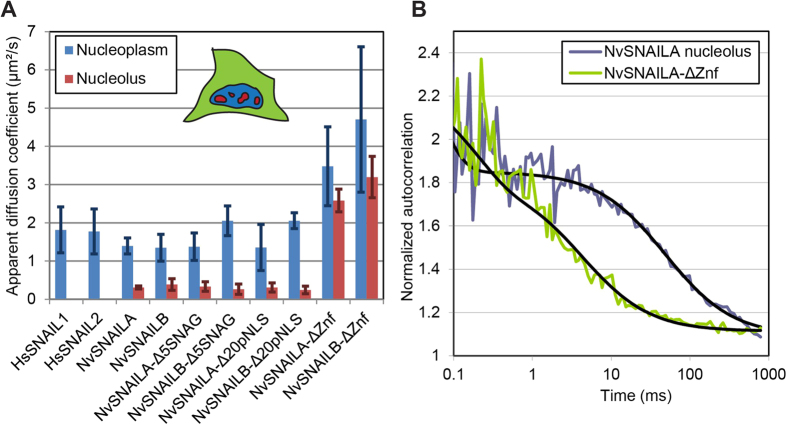
FCS Analysis of *Nv*SNAILA and B, their relative truncated variants in comparison with *Hs*Snail1 and 2. **A**) Apparent diffusion coefficients of the SNAIL constructs at different cellular locations, including the nucleoplasm, nucleolus as measured by FCS. The apparent diffusion coefficient of mTurquoise2 in the nucleoplasm was *D* = 23 ± 6 μm^2^/s. See main text for more details. Error bars are confidence intervals at 95% confidence level. **B**) Example of FCS autocorrelation plots measured for *Nv*SNAILA full length measured in the nucleolus and *Nv*SNAILA-ΔZnf measured in the nucleoplasm. Color lines denote the raw data, while black lines denote the fit of the curve to a diffusion model including dark-states (see Eq. 2 Methods).

Having identified different nuclear/sub-nuclear localization localization patterns of *Nv*SNAILA, B and *Hs*SNAIL1, 2 we decided to compare the same proteins analysing their mobility in the nuclear compartment by using Fluorescence Correlation Spectroscopy (FCS) (see Methods). Diffusion times were extracted from the auto-correlation curves and converted to apparent diffusion coefficients by using mTurquoise2 in PBS buffer as calibration (*τ*_*T*_ = 0.14 ms; *D* = 90 μm^2^/s, see Methods). The apparent diffusion coefficients were measured in different regions of the cell, including the nucleoplasm and nucleolus and represent a mix between free diffusion and transient binding (Fig. 8, supplemental Table S3).

For all full-length SNAIL constructs, an apparent diffusion coefficient ranging from 1–2 μm^2^/s was found in the nucleoplasm, which is significantly lower than mTurquoise2 in the nucleoplasm alone (*D* = 23 ± 6 μm^2^/s). These results indicate a similar mobility for the full-length proteins analysed in the nucleoplasm and more importantly they move slower than a free unbound protein moves in the same compartment.

### Mobility of the *Nv*SNAILA, B truncation mutants

In order to examine the role of SNAG domain, putative NLS and zinc-finger in protein mobility, we decided to measure the apparent diffusion coefficient for all the truncation mutants created finding that truncation of the SNAG domain or putative NLS do not significantly affect the mobility as *Nv*SNAILA/B-Δ5SNAG and *Nv*SNAILA/B-Δ20pNLS exhibited a similar mobility as the full length *Nv*SNAILA and *Nv*SNAILB (Fig. 8A, supplemental Table S3). Truncation of the zinc-finger domains does affect the mobility as both *Nv*SNAILA-ΔZnf and *Nv*SNAILB-ΔZnf have an apparent diffusion coefficient ranging from 3–5 μm^2^/s, which is about twice as mobile as the full length proteins.

### Characterization of the mobility in the nucleolus

To further characterize the mobility of *Nv*SNAILA and B, the apparent diffusion coefficient of the full length and respective mutant versions was measured also in the nucleolus. The mobility of full-length *Nv*SNAILA and *Nv*SNAILB constructs in the nucleolus showed a marked reduction compared to the nucleoplasm, with an apparent diffusion coefficient of about 0.3–0.4 μm^2^/s, which is about four fold lower (Fig. 8A,B, supplemental Table S3). *Nv*SNAILA/B-Δ5SNAG and *Nv*SNAILA/B-Δ20pNLS exhibited a similar reduction in mobility in the nucleolus, suggesting that truncation of the SNAG domain, while reducing nucleolar levels of NvSNAILA (Fig. 3) does not affect its mobility. However, truncation of the zinc-finger domains had a marked effect on the mobility in the nucleolus, for both *Nv*SNAILA-ΔZnf and *Nv*SNAILB-ΔZnf the apparent diffusion coefficient was about 2.5–3.5 μm^2^/s, which is about 10 fold higher than the full length constructs and only about 1.5 fold lower than in the nucleoplasm (Fig. 8, supplemental Table S3). For mTurquoise2 a similar reduction in mobility was found in the nucleolus compared to the cytoplasm, indicating that the bleach profile of these last two constructs and mTurquoise2 are comparable.

### FRAP analysis reveals fast exchange of *Nv*SNAILA between nucleolus and nucleoplasm

**Figure 9:**
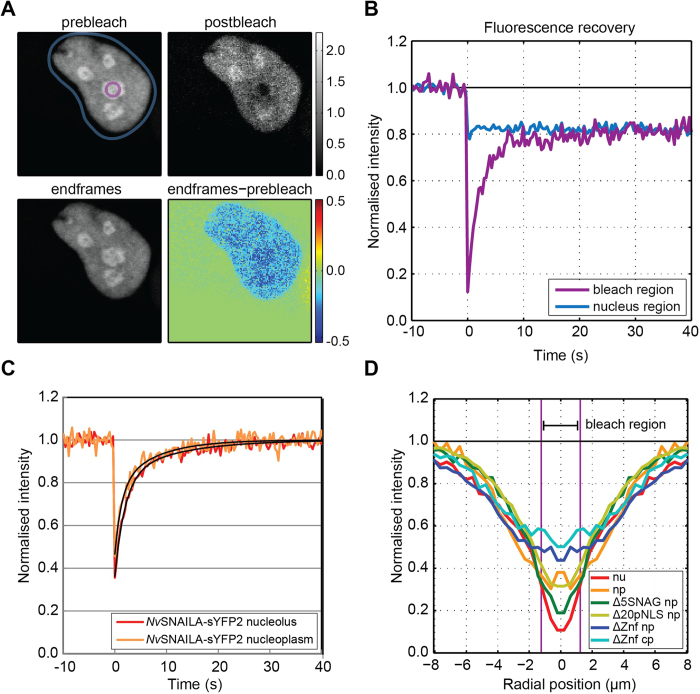
FRAP analysis of *Nv*SNAILA-sYFP2 constructs in different regions of HeLA cells. **A**) A cell transfected with *Nv*SNAILA-sYFP2 exhibits enriched nucleoli (prebleach) was bleached in a nucleolus (postbleach). After about 40 s the distribution of *Nv*SNAILA-sYFP2 in the cell is almost completely recovered (endframes, endframes-prebleach). **B**) Time course of the region containing the nucleus (blue) and the bleached circular region (purple), see main text for details. **C,D**) FRAP dynamics and radial profiles **C**) Removal of the FLIP effect from the recovery traces (*n* = 6 measurements) shows that fluorescence in the nucleolus and nucleoplasm is completely recovered within 40 s. The recovery curves were fitted with a double exponential, 1−*a*_1_ exp[−*t*/*τ*_1_]−*a*_2_ exp[−*t*/*τ*_2_], yielding, *a*_*1*_ = 0.46, *τ*_*1*_ = 1.98 s, *a*_*2*_ = 0.18 and *τ*_*2*_ = 11.12 s for the nucleolus and *a*_*1*_ = 0.31, *τ*_*1*_ = 1.31 s, *a*_*2*_ = 0.23 and *τ*_*2*_ = 7.37 s for the nucleoplasm. The nucleoplasm recovery is slightly faster than recovery in the nucleolus. **D**) Initial radial profiles just after the bleach shows that the relative mobility of all constructs fused to sYFP2 in different regions of the cell is consistent with the FCS measurements. Hence, more shallow gradients indicate higher mobility. The radial profiles show that *Nv*SNAILA in the nucleolus (nu) has a lower mobility than the mobility of *Nv*SNAILA, *Nv*SNAILA-Δ5SNAG and *Nv*SNAILA-Δ20pNLS in the nucleoplasm (np). The latter *Nv*SNAILA-ΔZnf shows similar initial profiles but appear to have a higher mobility compared to the other constructs in the nucleoplasm and in turn it has a lower mobility than *Nv*SNAILA-ΔZnf in the cytoplasm (cp).

The localisation analysis showed that *Nv*SNAILA is significantly enriched in the nucleolus and the FCS analysis indicated that it has a low apparent diffusion coefficient in the nucleolus compared to the nucleoplasm. The FCS analysis gives information about movement in and out of the detection volume; however it does not necessary give information about transport into and out of the nucleolus^37^. In order to study this process, a FRAP analysis was performed. For this experiment, the full length *Nv*SNAILA was fused to the yellow fluorescent protein sYFP2 (*Nv*SNAILA-sYFP2), which can be bleached more efficiently than mTurquoise2^28^. A circular region with a diameter of 2.484 μm was bleached for one second, after which recovery was monitored over a period of about forty seconds (see Methods). In Fig. 9A nucleolar enrichment of *Nv*SNAILA-sYFP2 is clearly visible in the averaged frame before bleaching (pre-bleach). Directly after bleaching the nucleolus is almost completely bleached (post-bleach) and after a period of about forty seconds the cell shows virtually the same distribution (end-frames and difference image end-frames minus pre-bleach, supplementary video S1). Because the nucleus is a confined compartment, the total fluorescence in the nucleus is reduced, which is apparent as a step change in the time trace of the nucleus region (Fig. 9B, blue trace); the part that is not recovered in the FRAP region (Fig. 9B, purple trace) can be completely explained by this loss of total nuclear fluorescence. If the total loss of nuclear fluorescence is removed from the FRAP region traces, residual traces can be obtained (Fig. 9C). These residual traces (*n* = 6 measurements) show that the nucleolus completely recovers and does not appear to have any significant immobile fraction and that the long term recovery in the nucleolus has a similar time course as recovery in the nucleoplasm. This suggests that exchange of *Nv*SNAILA-sYFP2 between the nucleolus and the nucleoplasm is relatively fast and that its long-term recovery is limited by diffusion from the nucleoplasm to the nucleolus and largely determined by the low diffusion coefficient in the nucleoplasm and the geometry of the nucleus. Further FRAP analysis was performed on *Nv*SNAILA-Δ5SNAG, *Nv*SNAILA-Δ20pNLS and *Nv*SNAILA-ΔZnf both in the nucleoplasm as well as the cytoplasm. The steepness of the radial profiles around the centre of the bleached area just after the bleach was determined (Fig. 9D), which represent an indication of the mobility of the different constructs and show that the FRAP results are in close agreement with the apparent diffusion coefficients that were obtained from the FCS analysis.

## Discussion

EMT is a crucial process occurring during both embryonic development and disease, such as tumour cell invasion. Snail family transcription factors have been found to play a key role in this context by down-regulating epithelial cadherins, which is an important hallmark of EMT. As any other transcriptional factor, its activity is regulated not just by potential cofactors, which might influence their binding to the DNA, but also by the presence/absence and specific sublocalization of the protein itself in the nucleus. The existence of specific signals such as the NLS allows the cellular system to regulate cellular levels of key proteins, by distributing those in specific cellular compartments corresponding to their function. In this study we have performed a detailed characterization of *Nematostella vectensis* transcription factors SNAILA and SNAILB using both mammalian cells and developing *Nematostella* embryos. We show differences in sub-nuclear distribution and mobility and compared these with their human homologs. *Nv*SNAILA is enriched at the Granular Component of human nucleoli and co-localises with GC markers after nucleolar disruption. Furthermore, nuclear localization, sub-nuclear distribution of both *Nv*SNAILA and *Nv*SNAILB seem to be dependent on the SNAG domain and the zinc-finger domains while the dynamics of both *Nv*SNAILA and B is specifically affected by the zinc-finger domains. According to our findings we draw four main conclusions:

1. *SNAIL domain analysis reveals several scenarios for the evolution of NvSNAIL proteins*. Results obtained from domain analysis (Fig. 1) suggest that despite *Nv*SNAILB often being identified as a 5 zinc-finger transcriptional factor^11^,^27^, it actually might have lost the functionality in one zinc-finger domain. Thus, at least two scenarios concerning the evolution of *Nv*SNAIL protein can be proposed (Fig. 2B). Among cnidarians *Nematostella vectensis* is the only species that doesn’t possess a clear four zinc-finger SNAIL factor, so in a first scenario *Nematostella* might once have had three snail genes (one additional with just four zinc-fingers) and subsequently lost one later on during evolution. On the other hand loss of the first zinc finger in one of the SNAIL transcription factors seems to be an event occurred several times during evolution and it might most likely have started with *Nematostella vectensis* within the cnidarians, where the structure of the first zinc-finger domain of *Nv*SNAILB is not fully conserved (Fig. 1C). Interestingly the placozoan snail gene groups closely with the other four zinc-finger snail genes of invertebrates (Fig. 2), suggesting those could be direct descendants.
2. *Zinc fingers are determinants for nucleolar localization of NvSNAILA.* We observed that substitution of the zinc-finger domains from *Nv*SNAILA with the *Nv*SNAILB zinc-finger domains leads to a marked reduction of nucleolar levels (Fig. 7), suggesting that fully conserved zinc-finger domains of *Nv*SNAILA are involved in nucleolar localization as it has been proposed at least once in literature for other zinc finger transcriptional factors^38^. This is further corroborated by the observation that substitution of the *Nv*SNAILB zinc-finger domains with the *Nv*SNAILA zinc-finger domains leads to a marked increase in nucleolar levels similar to the full length *Nv*SNAILA. Since *Nv*SNAILB lacks a fully conserved structure of the first zinc-finger domain at the C-terminus compared to *Nv*SNAILA while the SNAG domain at the N-terminus is highly conserved, there could be an involvement of the first zinc finger for nucleolar localization of *Nv*SNAILA. It has been shown before that NLS signals in SNAIL proteins are located in the second, third and fourth zinc-finger domains (corresponding to the first, second and third Zn finger for human SNAIL1). Furthermore, DNA binding is linked to the last 4 zinc-finger domains in SNAIL proteins having more than 4 zinc-finger domains, while the absence of the first zinc-finger domain seems to not affect neither localization nor DNA binding^22^,^25^. Thus, the first zinc-finger domain present in *Nv*SNAILA might have a role in protein-protein interaction stabilizing the protein either in a specific protein fold or by interaction with another cofactor, which may be linked to nucleolar localization.
3. Interestingly, we observed that the first 20 amino acids previously described as having a role in nuclear localization^21^,^22^ are not essential for nuclear localization of *Nv*SNAILA and *Nv*SNAILB, despite the presence of many charged residues at the N-terminus (Fig. 3). Instead, this region appears to affect nucleolar localization directly or indirectly; acting in combination with the zinc-finger domains in nucleolar localization, differently from what has been reported so far^39^. As such, a complete SNAG domain is required, but not sufficient for nucleolar localization of SNAILA. Indeed, truncation of the SNAG domain and the putative NLS do not affect nuclear localization, confirming the presence of a non-classical NLS within or around the zinc-finger domains as previously reported for higher organisms^40^. Interestingly, the same amino acids crucial for nuclear localization identified in the zinc fingers of vertebrate SNAILs are also conserved in *Nematostella vectensis* SNAILs (Fig. 1C). Specifically, mutation of the Arginine in position 220 and 224 of the fourth zinc finger of *Hs*SNAIL1 gives a localization^25^ very similar to the one observed for *Nv*SNAILA and B truncated of all (or just the last four) zinc-finger domains (Fig. 3), suggesting a conserved role for the same key amino acids between *Nematsotella* and human SNAILs for nuclear localization. Moreover, this indicates an ancestral and convenient strategy by which the cellular system used to distribute into the nucleus specific proteins possessing DNA binding domains, maybe through the interaction with a cellular transporter^25^. Intriguingly *Hs*SNAIL2 which possesses both the SNAG domain and all five zinc-finger domains does not localize into the nucleoli of HeLa cells with the same high specificity as *Nv*SNAILA, but exhibits levels more similar to *Nv*SNAILB, which lacks a fully conserved first zinc-finger domain. This might be due to the evolution of additional motifs as the ones included in the SLUG domain placed right before the zinc fingers and probably inhibiting folding/interaction required for nucleolar localization.
4. *Zinc fingers are determinants of NvSNAILs mobility. Nv*SNAILA, *NvS*NAILB, *Hs*SNAIL1 and *Hs*SNAIL2 show a mobility typical for transcriptional factors in the nucleoplasm^41^. Specifically and more importantly, in the nucleoplasm *Nv*SNAILA and B show apparent diffusion coefficient values comparable to the ones obtained for *Hs*SNAIL1 and 2, known for being transcriptional factors and of which a mobility measurement has been reported here for the first time. Interestingly we noted that truncations of both the SNAG domain alone or together with the putative NLS seem not to have any marked effect on the mobility, while truncation of all the zinc-finger domains leads to a slight increase in mobility (Figs 8A and 9D). From our data, indeed the zinc-fingers seem to be the main determinants for the mobility of *Nv*SNAILA and *Nv*SNAILB also in the nucleolus showing in this compartment a difference in mobility observed for mTq2 alone. Previous studies suggest that Snail gene-regulation activity in higher organisms is regulated by a mutual interaction with cofactors such as FOUR and HALF LIM protein2 (FHL2), LYSINE SPECIFIC DEMETHYLASE 1 (LSD1), p53 through the SNAIL Snag domain^12^,^23^,^22^,^24^,^42^, while the last four zinc-finger domains are involved in DNA binding. Therefore, given the high homology found in the zinc finger domains between human and *Nematostella* SNAILs the mobility analysis performed shows also that *Nv*SNAILA and B mobility seems to be strictly dependent on their interaction with the DNA instead of the interaction with a putative co-factor, at least in HeLa cells.
5. *NvSNAILA and B cellular distribution in both HeLa cells and Nematostella vectensis embryos suggests strong conservation between the two organisms.* The same specific nuclear localization was observed after expression of *Nv*SNAILA and B and their truncated variants in both HeLa cells and *Nematostella vectensis* embryos (Figs 3,6). Thus, HeLa cells might represent a complementary convenient model system to study the cellular behaviour of these proteins. Also the dynamics of the wild-type proteins studied is comparable since all of them exhibit similar values of apparent diffusion coefficients. Therefore, based on the zinc-finger homology relation, our data suggest that *Nv*SNAILA and *Nv*SNAILB could possibly bind DNA sequences, acting as transcriptional factors in HeLa cells in a similar way as *Hs*SNAIL1 and *Hs*SNAIL2. Further functional studies in *Nematostella or mammalian cells* concerning the mutant constructs presented in this work will be crucial for understanding the role of Snail transcriptional factors in this basal metazoan.

Finally, in this work we show for the first time that *Nv*SNAILA, differently from *Hs*SNAIL1 and 2 as well as other snail proteins characterized so far, exhibits pronounced enriched levels in HeLa cells nucleoli (Figs 3 and 5) as well as in the nucleoli of *Nematostella vectensis* embryos (Fig. 6), suggesting a potential function within this compartement. Furthermore, co-localisation analysis shows that *Nv*SNAILA co-localises with GC markers *Hs*B23 and *Hs*SENP3 even after nucleolus disruption and not with the DFC or FC (Fig. 5, S1). Although the NLS present in *Nv*SNAILA zinc fingers may accidently have converged during evolution to also have a NoLS function, the analysis obtained by FCS (Fig. 8) reveals low mobility within the nucleolar compartment directly linked to zinc-fingers, suggesting specific stabilisation or transport process of *Nv*SNAILA which might be the same that takes other nucleolar proteins into specific nucleolar regions. The presence of *Nv*SNAILA in the nucleoli might indicate another function for this protein in addition to acting as a classical transcriptional factor in the nucleoplasm. In fact, apart from being known as a “ribosome factory”^43^ the nucleolus has also been shown to be involved in cell cycle regulation. Evidence of a connection between nucleolus and cell cycle can be found in early literature^44^ but just recently this topic became of primary importance due to the relation with human diseases^45^. The association between ribosome synthesis and cell cycle regulation seems to occur through different proteins such as p53^46^, B23^47^ and cycline-dependent kinases^48^.

The enrichment of *Nv*SNAILA in the nucleoli could suggest that: i) being a zinc finger protein, *Nv*SNAILA might be involved during processing steps of rRNA synthesis, for instance during maturation of pre-rRNAs facilitating interactions between proteins and rRNA. Thus *Nv*SNAILA could have a similar role to *Hs*B23 or *Hs*SENP3^49^ in HeLa as well as in *Nematostella* embryos, perhaps being involved in Ribosomal subunit assembly together with other proteins. Notably, a relation between zinc fingers and nucleolar localization has been reported before and it may depend on the ability of the protein to bind nucleic acids^38^. On the other hand ii) it may be involved during stress or DNA damage, participating in the arrest of cell growth and induction of DNA repair, as proposed for other zinc fingers proteins found in the nucleolus^50^. Intriguingly, both of these cellular processes appear to be essential during mitosis occurring in embryogenesis^43^. Moreover, given that *Hs*SNAIL2 (but not *Hs*SNAIL1) also shows specific nucleolar localization, albeit it at far lower levels than *Nv*SNAILA (Fig. 3), a role for SNAIL proteins in the nucleolus may be conserved across evolution.

## Methods

### Plasmids used for HeLa cells analysis

cDNA for *Nematostella vectensis* SNAILA and B as well as the respective truncated variants were RT-PCR- amplified from *Nematostella vectensis* and cloned in pmTurquoise2, psYFP2, pmCherry (clontech-like) vectors providing a C-terminal mTurquoise2^28^, sYFP2 and mCherry tag. cDNA for human (*Hs*) SNAIL1 and 2 were RT-PCR- amplified from HeLa cells and cloned in pmTurquoise2 N1 (clontech-like) vectors providing a C-terminal mTurquoise2 tag. Coding DNA for human FIBRILLARIN (*Hs*FIB) was PCR- amplified from HeLa cells cDNA and cloned in psYFP2 C1 (clontech-like) vector providing an N-terminal sYFP2 tag. See supplementary dataset 1 all primer sequences.

A plasmid pmCherry (clontech-like) vector was used for quantification analysis. Hybrid genes were obtained by PCR-driven overlap extension as previously described^51^, and cloned in pmTurquoise2 N1 (clontech-like) vector providing an C-terminal mTurquoise2 tag. Plasmids encoding both Nucleolin (*Hs*NCL) (#28176) and sentrin-specific peptidase 3 (*Hs*SENP3) (#34554) fused to the GFP at their N-terminal in a C1 (clontech-like) vector were obtained from Addgene. Nucleophosmin (*Hs*B23) was obtained from B23-pCMV-DsRed-Express (Addgene, #34553) and cloned into pmTurquoise2 N1 (clontech-like) vector. See supplementary material for the primers used for each cloning step.

HeLa cervical cancer cells (ccl-2) were cultured and transfected as previously described^52^.

### Plasmids used for Nematostella vectensis embryos injections

The cDNA for *Nematostella vectensis* SNAILA as well as the respective truncated variants were subcloned into a vector suitable for *in vitro* transcription. We used the Gateway pSPE3-mCherry (for *Nv*SNAILA, B full length) and pSPE3-mVenus (for *Nv*SNAILA truncated variants: *Nv*SNAILA-Δ5SNAG, Δ20pNLS, ΔZnf and *Nv*SNAILB-ΔZnf) system^53^ for *in vitro* transcription vectors.

### RNA synthesis and microinjections

Linearization and RNA synthesis were carried out as previously described^54^. Uncleaved embryos were individually injected as previously described^54^ with the RNA of *Nv*SNAILA, B and the respective truncated variants at a final concentration of 300 ng/μl. After 10 hours at 25 °C the injected embryos from late cleavage stage were visualized by live microscopy.

### Hoechst staining

Embryos from late cleavage stages (10 hrs. post fertilization at room temperature) were fixed as previously described^55^. Embryos were then incubated in Hoechst (100 mg/ml in DMSO stock solution,Life Technologies) diluited 1:5000, in 1 ml PTw for 1 h at room temperature. Embryos were then cleared 2x with 50% glycerol in PBS and 2x 80% glycerol in PBS and mounted for visualization.

### Confocal microscopy and quantification in HeLa cells

Experiments were performed as previously described^52^. See supplementary Table S1 for the confocal microscopy settings. Based on three confocal microscopy images of the nucleolar marker *Hs*FIB (fused to sYFP2), free mCherry and the construct of interest (fused to mTurquoise2) transfected in HeLa cells (Fig. 4) we extracted the cytoplasm/nucleoplasm and nucleolus/nucleoplasm ratio. The cytoplasm/nucleoplasm ratio was calculated by dividing the median fluorescence value of cytoplasm by the median fluorescence value of the nucleoplasm, which was obtained using masks (see Fig. 4). We subsequently divided the ratio obtained for *Hs*FIB and the construct of interest by the cytoplasm/nucleoplasm ratio of mCherry in order to reduce variability due to cell geometry or cytoplasmic organelles. The ratios are therefore presented relative to mCherry, which can passively move into and out of the nucleus and is both present in the nucleoplasm as well as the cytoplasm, a value close to one means that the ratio is similar to mCherry.

We calculated the raw nucleolus/nucleoplasm ratio for each nucleolus by averaging the normalized pixel values for *Hs*FIB and the construct of interest in each nucleolar region using a nucleolar mask (see Fig. 4), and then calculated a weighted mean for each cell. Nucleoli are very dense structures and we assume that mCherry does not specifically bind there. Although mCherry was largely excluded from the nucleoli, we still measured about 50% residual fluorescence at the nucleolus (Fig. 4I,J), which represents the fraction (*f*) of non-specific localization and can be partly explained by fluorescence originating from the nucleoplasm. We therefore also calculated an adjusted nucleolus/nucleoplasm ratio^39^ by removing the fluorescence that comes from non-specific nucleolar localization. The adjusted nucleolus/nucleoplasm ratio can be calculated for each pixel using the following equation: 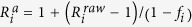. Here 1 − *f*_*i*_ represents the inaccessible volume fraction that is obtained from the normalized mCherry image *R*^*raw*^ represents the raw nucleolus/nucleoplasm ratio. Because of the noisy signals we calculate the average adjusted ratio using the least squares solution:
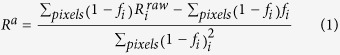
Where 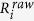 denotes the raw ratio for pixel *i* and *f*_*i*_ denotes the normalized mCherry fluorescence, which represents the freely accessible volume fraction, measured in the confocal volume. Adjusted ratios close to one represent a distribution similar to mCherry. See Fig. 4 legend for further details.

### DRB- Assay

Transfected HeLa cells were incubated at 37 °C and 5% CO_2_ with DRB (Sigma-Aldrich) added to the culture medium to the final concentration of 60 μM as previously described^35^. After 5 h the drug was washed away and cells were kept in standard medium for 20 and 60 min.

### Confocal microscopy in Nematostella embryos

See supplementary Table S1 for the confocal microscopy settings used.

### Fluorescence Correlation Spectroscopy (FCS)

FCS experiments were conducted approximately 12 hours after transfection. Measurements were performed on an inverted Fluoview 1000 laser scanning microscope (Olympus). The excitation light of a 440 nm 20 MHz pulsing laser diode (Picoquant), as controlled by a SepiaII laser driver unit (Picoquant), was attenuated 10 times by a neutral density filter. The light was guided via a D440/514/594 primary dichroic mirror (Chroma) through a water immersed 60x UPlanS-Apo objective (NA 1.2) into the sample. Cellular samples were grown on 24 mm round coverslips (Thermo scientific, Menzel-glaser) and fixed in AttoFluor sample holders (Invitrogen). The emission light was guided via a size-adjustable pinhole, set at 120 μm, through the Olympus detection box to the fibre output channel. The optical fibre was coupled to a custom-made detection box (Picoquant) containing PDM avalanche photodiodes (MPD). The light was guided into one of the MPDs where the light was filtered by a 475/45 emission filter (Chroma). The photon arrival times were recorded by a Picoharp 300 time-correlated single-photon counting system (Picoquant).

Samples were measured at room temperature (21 °C). The photon arrival times were recorded during 120 seconds by the SymPhoTime 5.23 (Picoquant) software. The size and shape of the observation volume was determined from FCS calibration measurements using purified mTurquoise2 in PBS as previously outlined^56^, and the estimated volume structure parameter was 8.

The diffusion time of the fusion proteins was analysed in FFS Data Processor 2.3 software (SSTC). Sections of raw data, lacking significant photobleaching and aggregation spikes, were autocorrelated and analysed using a one-component 3D diffusion model with correction for triplet/blinking kinetics^56^. The autocorrelation function was fitted to the following equation (see Fig. 8B for fitted examples):
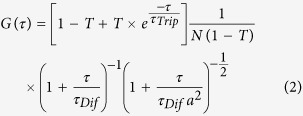
where *T* denotes the fraction of molecules in the dark state, *τ*_*Trip*_ the dark state relaxation time, *N* the number of molecules in the detection volume, *τ*_*Dif*_ the diffusion time constant and *a* the volume structure parameter. To improve fitting speed and quality, the lower bound of the diffusion time constant, *τ*_*Dif*_ was constraint to 200 μs the upper bound for the dark-state time constant, *τ*_*Trip*_ was constraint to 150 μs. Protein concentrations were determined using to the following equation:
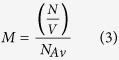
where *M* represents the concentration, *N*_*Av*_ denotes Avogadro’s number and the volume, *V* can be calculated using:
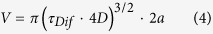
were *D* denotes the diffusion coefficient found for a free diffusing FP and is equal to 90 μm^2^/s^57^. The estimated concentration range under FCS conditions was 200–500 nM.

### Fluorescence recovery after photobleaching (FRAP)

Measurements were performed using the Olympus microscope as described for FCS. The 514 nm excitation line of an Argon laser (Omnichroma) was guided via a D458/515 primary dichroic mirror (Chroma). The emission light was guided to the internal PMT detector preceded by a 475/45 emission filter.

The cells transfected with constructs fused to the sYFP2 were imaged (128 × 128 pixels with xy dimensions of 207 nm) at a scan speed of 10 μs/pixel (0.331 s/frame). A circular region with a 6 pixel radius (1.242 μm) was bleached at 20% laser-power (approximately 100 μW) in tornado-scanning mode (bleaching from the centre outwards) for a bleach duration of 1 s. In total, 210 images were collected including 30 pre-bleach frames.

We determined the average fluorescence in the region of interest that was bleached and subtracted background fluorescence for each frame. We subsequently determined the fluorescence in the nucleus or the whole cell and fitted a double exponential with a step function to determine fluorescence bleaching due to scanning. The step was incorporated to account for loss of fluorescence in the confined nucleus or cell. In order to correct for scan bleaching the raw fluorescence profiles (bleach region and whole nucleus/cell) were subsequently normalized with the fitted double exponential without the step (see supplementary Fig. S2). Radial profiles were determined by calculating the average fluorescence in concentric rings, were each ring had a thickness of 207 nm. Regions outside the nucleus or cell were omitted in this calculation by using a mask.

## Additional Information

**How to cite this article**: Dattoli, A. A. *et al.* Domain analysis of the *Nematostella vectensis* SNAIL ortholog reveals unique nucleolar localization that depends on the zinc-finger domains. *Sci. Rep.* **5**, 12147; doi: 10.1038/srep12147 (2015).

## Supplementary Material

Supplementary Information

Supplementary Information
