# Supplementary material for: Domain analysis of the Nematostella vectensis SNAIL ortholog reveals unique nucleolar localization that depends on the zinc-finger domains
Source: Sci Rep. 2015 Jul 20;5:12147. doi: 10.1038/srep12147 (PMC4507178; doi:10.1038/srep12147)
Supplement: Supplementary Information [file srep12147-s2.pdf]

SUPPLEMENTARY INFORMATION

Domain analysis of the *Nematostella vectensis* SNAIL ortholog reveals unique nucleolar localization that depends on the zinc-finger domains

Ada A. Dattoli[1], Mark A. Hink[1, 2], Timothy Q. DuBuc[3], Bram J. Teunisse[1], Joachim Goedhart[1], Eric Röttinger [4, 5, 6] and Marten Postma[1*]

[1] Molecular Cytology, Swammerdam Institute for Life Sciences, University of Amsterdam Science Park 904, NL-1098 XH Amsterdam The Netherlands
[2] van Leeuwenhoek Centre for Advanced Microscopy (LCAM), University of Amsterdam, Science Park 904, NL-1098 XH Amsterdam The Netherlands
[3] The Whitney Laboratory for Marine Bioscience 9505 Ocean Shore Blvd, St. Augustine, FL 32080-8610
[4] Université Nice Sophia Antipolis, IRCAN, UMR 7284, 06107 Nice, France
[5] CNRS, IRCAN, UMR 7284, 06107 Nice, France
[6] INSERM, IRCAN, U1081, 06107 Nice, France

* Correspondence should be addressed to Dr Marten Postma, M.Postma@uva.nl (tel: +31 20 5257860)

Contents

Table S1	Confocal microscopy settings.
Table S2	Ratio of nucleolus fluorescence intensity over nucleoplasm fluorescence intensity (nu/np) and cytoplasm fluorescence intensity over nucleoplasm fluorescence intensity (cp/np) in HeLa cells.
Table S3	Apparent diffusion coefficients for the different constructs fused to mTq2 measured in HeLa cells at different cellular locations
Figure S1	Localisation analysis of *Nv*SNAILA in combination with *Hs*SENP3 and *Hs*NCL nucleolar markers after DRB-induced nucleolar disruption.
Figure S2	Normalization used in FRAP analysis, FRAP dynamics and radial profiles
Dataset 1	Primer sequences for cloning
Dataset 2	Accession numbers of SNAIL family members used for phylogenetic tree
Dataset 3	Alignment of the zinc-finger region used in constructing the phylogenetic tree
Dataset 4	E-values from SMART C2H2 domain predictions of the zinc-finger domain region for the sequences used in the phylogentic tree
Video S1	Video showing recovery after photobleaching of *Nv*SNAILA-sYFP2 in the nucleolus

Supplemental TABLE S1

Experiment performed	Microscope used	Excitation wavelenght	1st Dichroic mirror (Chroma)	Pinhole	Objective used	Colors detected	Filter
Co-Localization (HeLa cells)	Nikon A1	447nm (mTq2)	440/514	~30µm	60x, VC Plan-Apo (N.A. 1.4)	Cyan	482/35 BP
		514.5nm (sYFP2)				Yellow	550/40 BP
Co-Localization (quantification and DRB assay)	Nikon A1	447nm (mTq2)	457/514/561	~30µm	60x, VC Plan-Apo (N.A. 1.4)	Cyan	482/35 BP
		514.5nm (sYFP2)				Yellow	550/40 BP
		561nm (mCherry)				Red	585 LP
Co-Localization DRB assay	Nikon A1	488nm (mTq2)	405/488/561	~30µm	60x, VC Plan-Apo (N.A. 1.4)	Green	525/30 BP
		561nm (mCherry)				Red	585 LP
Co-Localization (*Nemve* embryos)	LSM 510	514.5nm (sYFP2)	514/561	~30µm	100x, VC Plan-NeoFluar (N.A. 1.3)	Yellow	525/30 BP
		561nm (mCherry)				Red	585 LP
Co-Localization (*Nemve* embryos)	LSM 510	351nm (Hoechst)	UV/488/543/633	~30µm	100x, Zeiss Plan-NeoFluar (N.A. 1.3)	Blue	385 LP

Confocal Microscopy settings.

Supplemental TABLE S2

**Construct/ratio**	**adj. R_{nu-np}**	**raw R_{nu-np}**	**adj. R_{cp-np}**
***Hs*FIB**	11.06 ± 1.41 (152)	5.21 ± 0.62 (152)	
***Hs*SNAIL1**	0.27 ± 0.09 (31)	0.72 ± 0.03 (31)	0.17 ± 0.04 (31)
***Hs*SNAIL2**	0.52 ± 0.09 (32)	0.82 ± 0.03 (32)	0.22 ± 0.05 (31)
***Nv*SNAILA**	2.46 ± 0.25 (27)	1.59 ± 0.10 (27)	0.13 ± 0.05 (27)
***Nv*SNAILA-Δ5SNAG**	0.45 ± 0.09 (12)	0.77 ± 0.04 (12)	0.25 ± 0.12 (12)
***Nv*SNAILA-Δ20pNLS**	0.44 ± 0.09 (14)	0.77 ± 0.04 (14)	0.08 ± 0.03 (14)
***Nv*SNAILA-ΔZnf**	0.32 ± 0.04 (13)	0.72 ± 0.02 (13)	0.96 ± 0.04 (13)
***Nv*SNAILB**	0.90 ± 0.15 (14)	0.96 ± 0.07 (14)	0.07 ± 0.03 (14)
***Nv*SNAILB-Δ5SNAG**	0.63 ± 0.10 (14)	0.83 ± 0.05 (14)	0.05 ± 0.02 (14)
***Nv*SNAILB-Δ20pNLS**	0.59 ± 0.08 (14)	0.83 ± 0.03 (14)	0.16 ± 0.12 (14)
***Nv*SNAILB-ΔZnf**	0.29 ± 0.03 (16)	0.68 ± 0.03 (16)	1.00 ± 0.04 (16)
***Nv*SNAILBNt-*Nv*SNAILAZnf**	0.92 ± 0.12 (15)	0.97 ± 0.05 (15)	0.04 ± 0.02 (15)
***Nv*SNAILANt-*Nv*SNAILBZnf**	2.53 ± 0.25 (14)	1.65 ± 0.10 (14)	0.07 ± 0.03 (14)
**mCherry***		0.62 ± 0.01 (275)	0.89 ± 0.01 (275)

Nucleolus/nucleoplasm ratios and normalized cytoplasm/nucleoplasm ratios measured in HeLa cells for different SNAIL proteins and SNAIL mutants.

*mCherry nucleolus/nucleoplasm and cytoplasm/nucleoplasm ratios are raw unadjusted ratios

Supplemental TABLE S3

Diffusion coefficients (µm²/s)	Nucleoplasm	Nucleolus	Cytoplasm
**mTq2**	22.56 ± 5.68 (11)	8.92 ± 2.17 (12)	17.66 ± 4.26 (10)
***Hs*SNAIL1**	1.82 ± 0.60 (9)	-	-
***Hs*SNAIL2**	1.77 ± 0.59 (9)	-	-
***Nv*SNAILA**	1.40 ± 0.21 (12)	0.31 ± 0.04 (19)	-
***Nv*SNAILB**	1.35 ± 0.35 (15)	0.39 ± 0.15 (7)	-
***Nv*SNAILA-Δ5SNAG**	1.38 ± 0.36 (10)	0.33 ± 0.13 (11)	-
***Nv*SNAILB-Δ5SNAG**	2.05 ± 0.39 (12)	0.26 ± 0.13 (10)	-
***Nv*SNAILA-Δ20pNLS**	1.35 ± 0.60 (11)	0.31 ± 0.12 (13)	-
***Nv*SNAILB-Δ20pNLS**	2.05 ± 0.21 (8)	0.24 ± 0.10 (11)	-
***Nv*SNAILA-ΔZnf**	3.48 ± 1.03 (9)	2.58 ± 0.30 (13)	4.48 ± 1.90 (9)
***Nv*SNAILB-ΔZnf**	4.70 ± 1.90 (11)	3.20 ± 0.54 (11)	6.50 ± 1.80 (11)

Diffusion coefficients for different SNAIL proteins and SNAIL mutants measured in different cellular compartments of HeLa cells.

Supplemental FIGURE S1

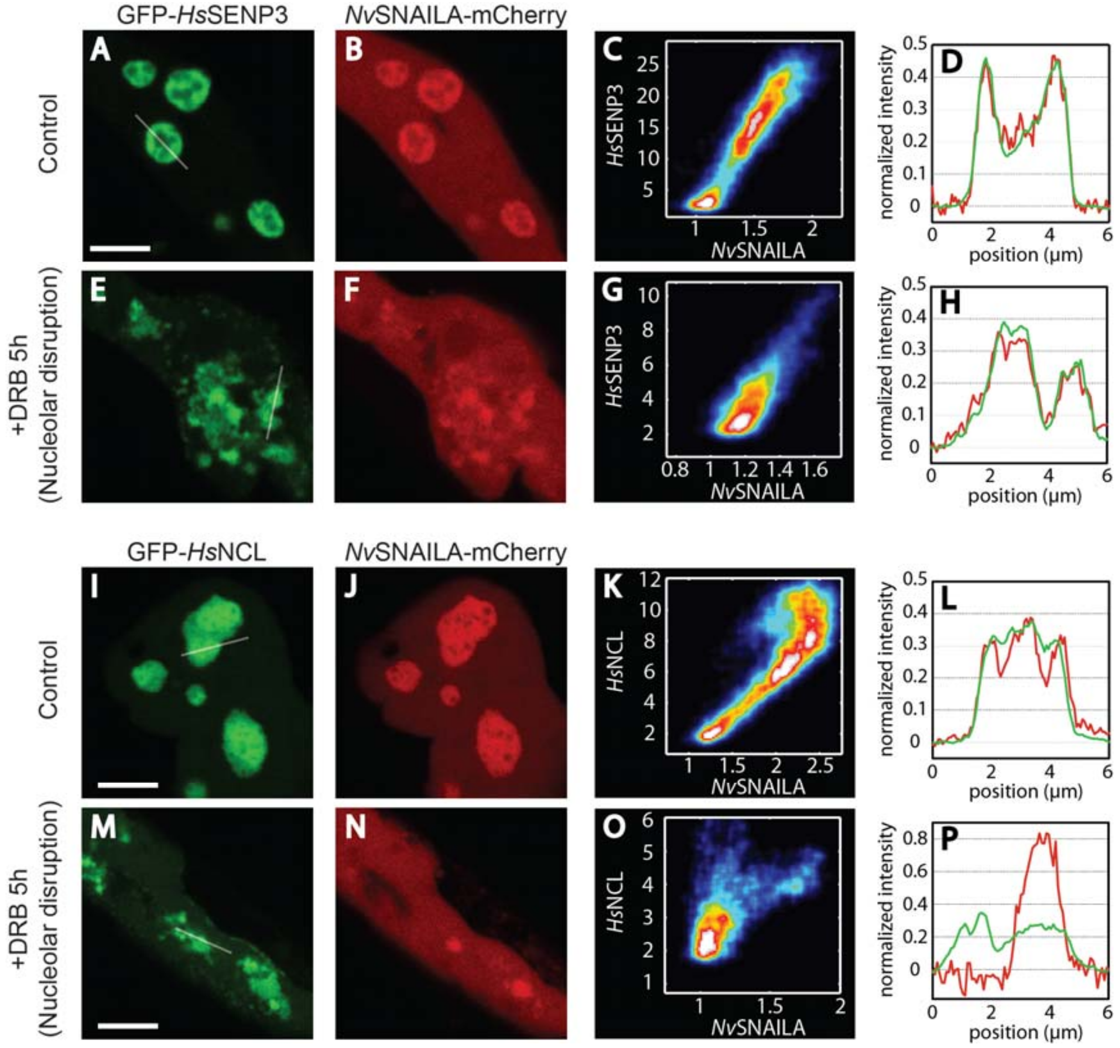

Figure S1. Localisation analysis of *Nv*SNAILA in combination with *Hs*SENP3 and *Hs*NCL nucleolar markers after DRB-induced nucleolar disruption. A-B) Control HeLa cells showing the specific nucleolar localization of *Hs*SENP3 and *Nv*SNAILA. **C**) Smooth Scatter-Plot for the couple *Nv*SNAILA- *Hs*SENP3 obtained by plotting the pixel intensity of each point from both channel in a region defined by a line in **A**. **D**). Plot of the fluorescence intensity of the two protein cosidered obtained in a nucleolar sub-region (white line in **A**). **E-F**) Separation of the DFC from the GC due to 5h DRB treatment. **G**) Smooth Scatter-Plot for the couple *Nv*SNAILA-*Hs*SENP3 obtained by plotting the pixel intensity of each point from both channel in a region defined by a line in **E**. **H**). Plot of the fluorescence intensity of the three protein cosidered obtained in a nucleolar sub-region (white line in **E**). **I-J**) Control HeLa cells showing the specific nucleolar localization of *Hs*NCL and *Nv*SNAILA. **K**) Smooth Scatter-Plot for the couple *Nv*SNAILA- *Hs*NCL obtained by plotting the pixel intensity of each point from both channel in a region defined by a line in **I**. **L**). Plot of the fluorescence intensity of the two protein cosidered obtained in a nucleolar sub-region (white line in **I**). **M-N**) Separation of the DFC from the GC due to 5h DRB

treatment. **O**) Smooth Scatter-Plot for the couple *Nv*SNAILA- *Hs*NCL obtained by plotting the pixel intensity of each point from both channel in a region defined by a line in **M**. **H**). Plot of the fluorescence intensity of the three protein cosidered obtained in a nucleolar sub-region (white line in **M**). Scale bar 10 µm.

Supplemental FIGURE S2

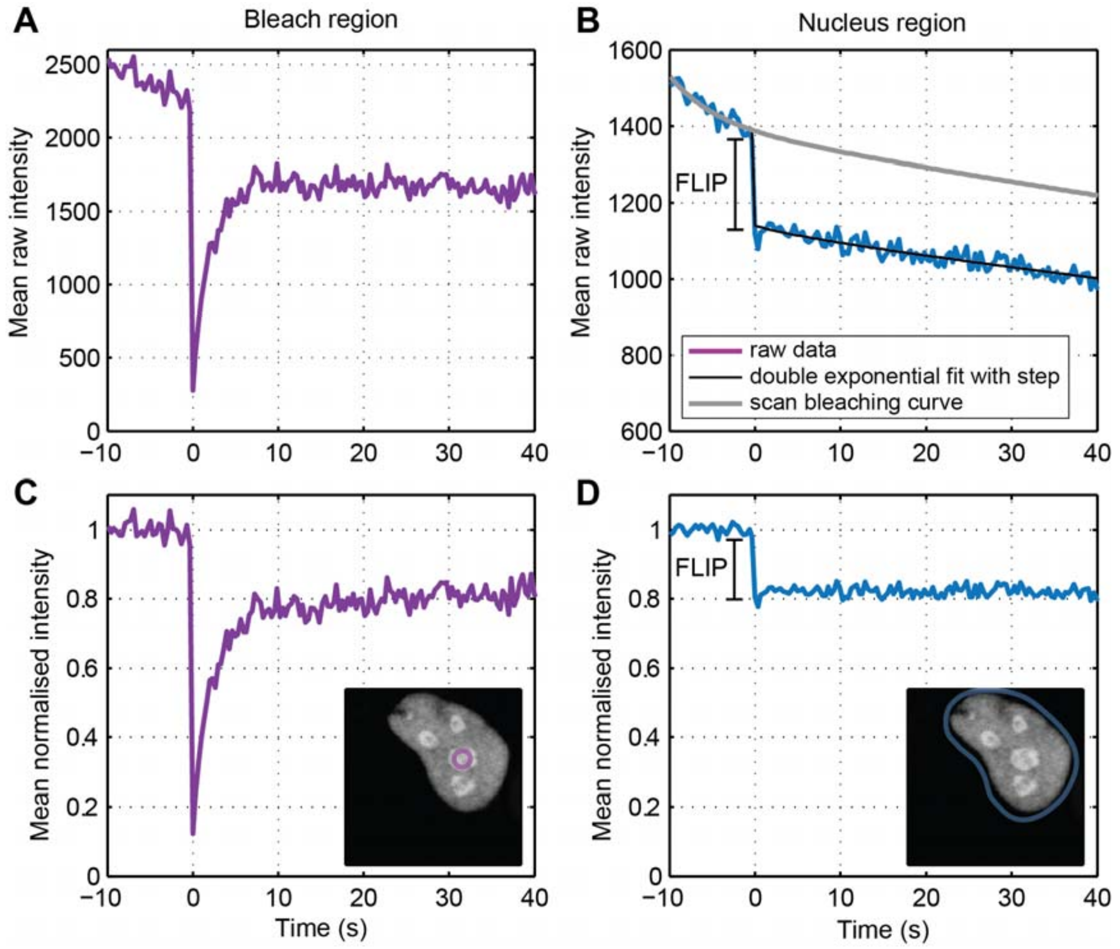

Figure S2. Normalization used in FRAP analysis. **A**) Raw trace of the mean fluorescence in the circular region that bleached a nucleolus. **B**) Raw trace of the mean fluorescence in the region that encompasses the whole nucleus. Because the nucleus is a confined region, loss of total fluorescence is apparent as a step (FLIP). A double exponential with a step was fitted (solid black line) to this trace and by removing the step after the fit an estimate was obtained for scan bleaching (solid grey line). **C-D**) In order to correct for scan bleaching, the raw traces of the circular region and the nucleus region were divided by the estimated scan bleaching curve and were subsequently normalized to unity using the mean pre-bleach values.

DATASET 1: Primer sequences

Constructs used in this study

Construct	Forward	Reverse
*Nv*SnailA	ATGCCCCGCTCGTTTCT	TCCTTGTGACGGGCAGCCCG
*Nv*SnailA-Δ5SNAG	ATGCTAGTCAAGAAAAC	TCCTTGTGACGGGCAGCCCG
*Nv*SnailA-Δ20pNLS	ATGGGGCCGGTAATGAC	TCCTTGTGACGGGCAGCCCG
*Nv*SnailA-ΔZnf	ATGCCCCGCTCGTTTCT	TCGCGATTCCTCCGCGGTTA
*Nv*SnailB	ATGCCGAGGTCCTTCCT	CCGCAGAGATTTTGCCGACACA
*Nv*SnailB-Δ5SNAG	ATGCTGGTGAAGACTAAG	CCGCAGAGATTTTGCCGACACA
*Nv*SnailB-Δ20pNLS	ATGATAATCCCTTCAAGA	CCGCAGAGATTTTGCCGACACA
*Nv*SnailB-ΔZnf	ATGCCGAGGTCCTTCCT	CCGGCAGGTGCTTCGCTCGTTA
*Hs*SNAIL1	ATGCCGCGCTCTTTCCT	GCGGGGACATCCTGAGCAGC
*Hs*SNAIL2	ATGCCGCGCTCCTTCCTGGTC	GTGTGCTACACAGCAGCCAGA
*Hs*FIB	ATGAAGCCAGGATTCAGT	TCAGTTCTTCACCTTGGGGGGTG
*Nv*SnailB-ΔZnfI	ATGCCGAGGTCCTTCCT	GTGGAATTGTTGATGTTT

Hybrids were obtained using the following primer pairs named a, b, c, d as indicated in the paper (Heckman & Pease, 2007)

	*Nv*SNAILANt-*Nv*SNAILBZnf	*Nv*SNAILBNt-*Nv*SNAILAZnf
a	ATGCCCCGCTCGTTTCTAGTC	ATGCCGAGGTCCTTCCT
b	TGGGCACTGGTGTTTGGGTC	ATGGCATTGGAGTTTGGCAG
c	GACCCAAACACCAGTGCCCA	CTGCCAAACTCCAATGCCAT
d	CCGCAGAGATTTTGCCGACACA	TCCTTGTGACGGGCAGCCCG

Primers for race PCR of Trichoplax SNAIL

	Forward	Reverse
*Ta*SNAILA-1	CGGTAAAGCGTTCAGTCGGC	CGCATCCAAATGGTTTTTCTCCAG
*Ta*SNAILA-2	CTGGAGAAAAACCATTTGGATGCG	CCGACTGAACGCTTTACCGC

Dataset 2: Accession numbers of SNAIL family members used for phylogenetic tree

Species	Acronym	Common name	Protein name (NCBI)	Name used	Changed name	Accession no.	Database
Caenorhabditis elegans	*Ce*	nematode	ces1	*Ce* SCRATCH	*x*	AAF01678	NCBI
Helobdella robusta	*Hr*	leech	snail1	*Hr* SNAIL1		AAL05564	NCBI
Patella vulgata	*Pv*	limpet	snail1	*Pv* SNAIL1		AAL06240	NCBI
		limpet	snail2	*Pv* SNAIL2		AAL12167	NCBI
Drosophila melanogaster	*Dm*	fruitfly	snail	*Dm* SNAIL		S06222	NCBI
		fruitfly	escargot	*Dm* ESCARGOT		S33639	NCBI
		fruitfly	worniu	*Dm* WORNIU		AAF12733	NCBI
		fruitfly	scratch	*Dm* SCRATCH		AAA91035	NCBI
		fruitfly	scratch-like1	*Dm* SCRATCH1-like		AAF47818	NCBI
		fruitfly	scratch-like2	*Dm* SCRATCH2-like		AAF47394	NCBI
Halocynthia roretzi	*Hr*	ascidia	Snail	*Haro* SNAIL		BAA75811	NCBI
Ciona intestinalis	*Ci*	ascidia	Snail	*Ci* SNAIL		AAB61226	NCBI
Branchiostoma floridae	*Bf*	amphioxus	Snail	*Bf* SNAIL		AAC35351	NCBI
		amphioxus	Snail-like	*Bf* SCRATCH	*x*	XP_002604602	NCBI
Takifugu rubripes	*Tr*	pufferfish	snail1	*Tr* SNAIL1		XP_003973759	NCBI
		pufferfish	snail2	*Tr* SNAIL1-like	*x*	XP_003963401	NCBI
		pufferfish	Snail3	*Tr* SNAIL3		XP_003963682	NCBI
		pufferfish	slug	*Tr* SLUG		XP_003975308	NCBI
Danio rerio	*Dr*	zebrafish	snail1	*Dr* SNAIL1		CAA52795	NCBI
		zebrafish	snail2	*Dr* SNAIL1-like	*x*	AAA87196	NCBI
		zebrafish	slug	*Dr* SLUG		NP_001008581	NCBI
		zebrafish	scratch1	*Dr* SCRATCH1		NP_001014369	NCBI
		zebrafish	scratch2	*Dr* SCRATCH2		NP_998802	NCBI
		zebrafish	Snail3	*Dr* SNAIL3		NP_001070853	NCBI
Xenopus laevis	*Xl*	african toad	Snail1	*Xl* SNAIL1		NP_001079925	NCBI
		african toad	Snail2	*Xl* SNAIL2		NP_001079751	NCBI
Silurana tropicalis	*St*	Western frog	Snail1	*St* SNAIL1-like	*x*	NP_989267	NCBI
		Western frog	Snail3	*St* SNAIL1	*x*	NP_001120305	NCBI
		Western frog	scratch1-like	*St* SCRATCH1-like		XP_002944038	NCBI
Gallus gallus	*Gg*	chicken	Snail1	*Gg* SNAIL1		NP_990473	NCBI
		chicken	Snail2	*Gg* SNAIL2		XP_419196	NCBI
		chicken	Scratch2	*Gg* SCRATCH2		NP_001166993	NCBI
Mus musculus	*Mm*	mouse	Snail	*Mm* SNAIL1		NP_035557	NCBI
		mouse	Slug	*Mm* SNAIL2	*x*	NP_035545	NCBI
		mouse	Smuc	*Mm* SNAIL3	*x*	NP038942	NCBI
		mouse	Scratch	*Mm* SCRATCH1		NP_570963	NCBI
		mouse	Scratch2	*Mm* SCRATCH2		NP_001153882	NCBI
Homo sapiens	*Hs*	human	Snail1	*Hs* SNAIL1		NP_005976	NCBI
		human	Snail2	*Hs* SNAIL2		NP_003059	NCBI
		human	Snail3	*Hs* SNAIL3		NP_840101	NCBI
		human	SCRATCH1	*Hs* SCRATCH1		NP_112599	NCBI
		human	SCRATCH2	*Hs* SCRATCH2		NP_149120	NCBI
Nematostella Vectensis	*Nv*	sea anemone	SnailA	*Nv* SNAILA		AAR24456	NCBI
		sea anemone	SnailB	*Nv* SNAILB		AAR24457	NCBI
		sea anemone	Scratch-like	*Nv* SCRATCH	*x*	XP_001632243	NCBI
Acropora millepora	*Am*	coral	Snail-2	*Am* SNAIL2		ACO55053	NCBI
		coral	znf-like	*Am* SNAIL1	*x*	AAS99630	NCBI
Trichoplax adhaerens	*Ta*	placozoa	Snail	*Ta* SNAIL1		XP_002108251	NCBI
		placozoa	znf-like	*Ta* SCRATCH	*x*	XP_002108252	NCBI
Cupiennius salei	*Cs*	spider	Snail	*Cs* SNAIL1		CAE00182	NCBI
Podocoryna carnea	*Pc*	jellyfish	Snail	*Pc* SNAIL1	*x*	CAD21523	NCBI
Hydra magnipapillata	*Hm*	hydra	zf-like 426	*Hm* SNAIL1	*x*	XP_002162096	NCBI
Strongylocentrotus purpuratus	*Sp*	sea urchin	Snail	*Sp* SNAIL		NP_999825	NCBI
		sea urchin	Snail2-like	*SpS* CRATCH1	*x*	XP_785413	NCBI
		sea urchin	Scratch-2-like	*Sp* SCRATCH2-like		XP_003724407	NCBI
Daphnia pulex	Dp	water flea	Escargot	*Dp* ESCARGOT		EFX80938	NCBI
			Snail-like	*Dp* SCRATCH-LIKE	x	EFX69682.1	NCBI
			Snail-like	*Dp* SCRATCH1	x	EFX77680.1	NCBI
Anolis carolinensis	Ac	anole lizard	Snail2-like	*Ac* SNAIL2-like		XP_003223554	NCBI
Orcinus orca	Oo	killer whale	Snail3	*Oo* SNAIL3		XP_004280112	NCBI
Meleagris gallopavo	Mg	wild Turkey	Snail-like	*Mg* SNAIL1	x	XP_003212214	NCBI
Saccoglossus kowalevskii	Sk	acorn worms	Snail	*Sk* SNAIL		NP_001158460	NCBI
Ornithorhynchus anatinus	Oa	platypus	Snail-like	*Oa* SNAIL1	x	XP_001507897.2	NCBI
Achaearanea tepidariorum	At	common house spider	Snail	*At* SNAIL		BAD44735	NCBI
Crassostrea gigas	Cg	pacific oyster	Escargot	*Cg* ESCARGOT		EKC40170	NCBI
Trichinella spiralis	Ts	pork worm	Escargot	*Ts* ESCARGOT		XP_003371527.1	NCBI
Acropora digitifera	Ad	coral	Snail	*Ad* SNAIL1	x	v1.11963	OSD
		coral	Snail-like2	*Ad* SCRATCH	x	v1.02984	OSD
		coral	Snail-like	*Ad* SNAIL2	x	v1.008519	OSD

*OSD=Organism Specific genome Database

Dataset 3: Alignment of the zinc-finger region used in constructing the phylogenetic tree

```
SpSCRATCH1(Sea_urchin)/1-139         GHSCPECRKSYSTSSN--LAR-HRQ-THR------------SVTDQKARKCPHCEKVYVSMPALSMHIRTHKLGCKCHICGKCFSRPWLLQGHIRTHTGERPFSCPQCGKAFADKSNLRAHVQTHSTIKPYVCQKCN-KAFALKSYLYKHVESACY
DmSCRATCH-like2_(Fruitfly)/1-139     EHICPECGKKYSTSSN--LAR-HRQ-THR------------SIMDKKARHCPYCEKVYVSMPAYSMHVRTHNQGCECQFCGKRFSRPWLLQGHIRTHTGEKPFKCGVCEKAFADKSNLRAHIQTHSNTKPHTCARCG-KAFALKSYLYKHEESSCM
DpSCRATCH-like_(Water_flea)/1-139    GHECPDCGKRYSTSSN--LAR-HRQ-THR------------SPADQKARRCPHCDKVYVSVPAFSMHVRTHSQGCKCPYCGKSFSRPWLLQGHIRTHTGEKPFTCQICEKAFADKSNLRAHIQTHSNLKPFTCQRCG-KAFALKSYLYKHEESSCM
CeSCRATCH_(Nematode)/1-138           RCVCDKCGKSYATTSN--LSR-HKQ-THR------------ALDSPHAKQCPHCDRVYVSMPALSMHILTHNASHECNVCGKRFSRLWLLQGHLRSHTGLRPFSCAHCGKSFADRSNLRAHMLTHTGDKRFECDKCG-RRFALRAYLNRHLET-CK
NvSCRATCH_(sea_anemone)/1-139        RYVCAECGKSYATSSN--LSR-HKQ-THR------------SLDGKLAKRCHQCGKAYVSMPALAMHVLTHKLLHKCEICGKSFSRPWLLQGHKRSHTGERPYSCPECNKAFADRSNLRAHLQTHNPLKQYKCERCD-RTFALKSYLNKHLESACS
AdSCRATCH_(Stony_coral)/1-139        RYICAECGKSYATSSN--LSR-HKQ-THR------------SLDSKLAKRCEHCGKAYVSMPALAMHILTHKLHHKCDVCGKAFSRPWLLQGHMRSHTGERPFMCPECNKAFADRSNLRAHMQTHSPLKQFKCNRCD-RTFALKSYLNKHAESACA
TaSCRATCH/1-139                      RYTCAECGKQYATSSN--LSR-HKQ-THR------------SLDGQLARRCKYCDKAYVSMPALAMHVLTHELAHKCNICGKAFSRSWLLQGHMRSHTGEKPYACATCNKRFADRSNLRAHMQTHSSVKSFQCKNCG-KSFALKSYLNKHAESGCC
DpSCRATCH1_(Water_flea)/1-139        RYVCGECGKAYATSSN--LSR-HKQ-THR------------SLESGCARTCPTCGKAYVSMPALSMHLLTHALSHVCPVCSKAFSRPWLLQGHMRSHTGEKPYGCAHCGKAFADRSNLRAHMQTHSATKSHHCGKCH-KAFALKSYLNKHLESSCF
DmSCRATCH_(Fruitfly)/1-137           KYTCSECGKQYATSSN--LSR-HKQ-THV--------------DSQSAKKCHTCGKAYVSMPALAMHLLTHKLSHSCGVCGKLFSRPWLLQGHLRSHTGEKPYGCAHCGKAFADRSNLRAHMQTHSVDKNFECKRCH-KTFALKSYLNKHLESACL
DmSCRATCH1-like_(Fruitfly)/1-139     CYKCCECGKQYATSSN--LSR-HKQ-THR------------SLDSQSAKKCNTCGKAYVSMPALAMHLLTHKLSHSCDICGKLFSRPWLLQGHLRSHTGEKPYACVHCGKAFADRSNLRAHMQTHSGDKNFKCHRCN-KTFALKSYLNKHLESACL
SpSCRATCH-like_(Sea_urchin)/1-139    KYKCNECGKQYATSSN--LSR-HKQ-THR------------SLDSHLAKKCEVCNKVYVSMPALAMHVLTHNLKHKCNVCHKSFSRPWLLQGHMRSHTGEKPFGCAHCGKAFADRSNLRAHMQTHSAYKNYKCKRCD-KSFALKSYLNKHYESACF
BfSCRATCH_(Amphioxus)/1-139          RYTCNECGKQYATSSN--LSR-HKQ-THR------------PLDSKLAKTCPTCGKVYVSMPALSMHVLTHQLSHKCDICNKAFSRPWLLQGHMRSHTGEKPFGCAHCGKAFADRSNLRAHMQTHSAFKQYKCKRCN-KSFALKSYLNKHYESACF
GgSCRATCH2_(Chicken)/1-139           RHSCPECGKTYATSSN--LSR-HKQ-THR------------SLDSKMARKCPTCGKAYVSMPALAMHVLTHNLKHKCDVCGKAFSRPWLLQGHMRSHTGEKPFGCSHCGKAFADRSNLRAHMQTHSAFKHYKCKQCE-KTFALKSYLNKHYESACF
DrSCRATCH2_(Zebrafish)/1-139         RHTCNECGKTYATSSN--LSR-HKQ-THR------------SLDSKMARKCPTCDKVYVSMPALAMHILTHDLKHKCHVCSKAFSRPWLLQGHMRSHTGEKPFACAHCGKAFADRSNLRAHMQTHSAFKHYSCKRCN-KTFALKSYLNKHYESACF
StSCRATCH1-like_(Western_frog)/1-139 RHSCSECGKSYATSSN--LSR-HKQ-THR------------SLDSKLAKKCPTCGKVYVSMPAMAMHLLTHDLKHKCETCGKAFSRPWLLQGHMRSHTGEKPFGCAHCGKAFADRSNLRAHMQTHSAFKHYKCKRCS-KTFALKSYLNKHYESACF
DrSCRATCH1_(Zebrafish)/1-139         RHTCNECGKTYATSSN--LSR-HKQ-THR------------SLDSKMAKKCPTCGKVYVSMPAMAMHLLTHDLKHKCDICGKAFSRPWLLQGHMRSHTGEKPFGCAHCGKAFADRSNLRAHMQTHSAFKHFKCKRCN-KTFVLKSYLNKHYESACF
HsSCRATCH1_(Human)/1-139             RHACGECGKTYATSSN--LSR-HKQ-THR------------SLDSQLARRCPTCGKVYVSMPAMAMHLLTHDLRHKCGVCGKAFSRPWLLQGHMRSHTGEKPFGCAHCGKAFADRSNLRAHMQTHSAFKHFQCKRCK-KSFALKSYLNKHYESACF
MmSCRATCH1_(Mouse)/1-139             RHACGECGKTYATSSN--LSR-HKQ-THR------------SLDSQLARRCPTCGKVYVSMPAMAMHLLTHDLRHKCGVCGKAFSRPWLLQGHMRSHTGEKPFGCAHCGKAFADRSNLRAHMQTHSAFKHFQCKRCK-KSFALKSYLNKHYESACF
HsSCRATCH2_(Human)/1-139             RHACAECGKTYATSSN--LSR-HKQ-THR------------SLDSQLARKCPTCGKAYVSMPALAMHVLTHNLRHKCGVCGKAFSRPWLLQGHMRSHTGEKPFGCAHCGKAFADRSNLRAHMQTHSAFKHYRCRQCD-KSFALKSYLHKHCEAACA
MmSCRATCH2_(Mouse)/1-139             RHACAECGKMYATSSN--LSR-HKQ-THR------------SLDSQLARKCPTCGKAYVSMPALAMHVLTHNLRHKCGVCGKAFSRPWLLQGHMRSHTGEKPFGCAHCGKAFADRSNLRAHMQTHSAFKHYRCRQCD-KSFALKSYLHKHCEAACV
DmSNAIL_(Fruitfly)/1-143             RFKCDECQKMYSTSMG--LSK-HRQ-FHCP--------AAECNQEKKTHSCEECGKLYTTIGALKMHIRTHTLPCKCPICGKAFSRPWLLQGHIRTHTGEKPFQCPDCPRSFADRSNLRAHQQTHVDVKKYACQVCH-KSFSRMSLLNKHSSSNCT
AtSNAIL_(Common_house_spider)/1-139  RYFCSACSKNYATYSG--LSR-HKQ-FHC------------ITLEKKAFNCKYCDKIYVSLGALKMHIRTHTLPCKCNLCGKAFSRPWLLQGHIRTHTGEKPFACPHCSRAFADRSNLRAHLQTHSDVKKYSCNACS-KTFSRMSLLLKHNDGGCG
CsSNAIL_(Spider)/1-139               RYQCPDCNKSYSTYSG--LSK-HRL-MHC------------ATQAKKSFGCKYCDKVYVSLGALKMHIRTHTLPCKCKLCGKAFSRPWLLQGHIRTHTGEKPFSCPHCSRAFADRSNLRAHLQTHSEVKKYRCKTCS-KTFSRMSLLLKHEDGGCA
DpESCARGOT_(Water_flea)/1-142        RYQCPDCHKSYSTYCG--LTK-HQE-LHC---------AAQQAANKKSFSCKHCEKVYVSLGALKMHIRTHTLPCKCLLCGKAFSRPWLLQGHIRTHTGEKPFSCPHCSRSFADRSNLRAHLQTHSDVKRYSCKSCG-KTFSRMSLLSKHEDGGCG
DmWORNIU_(Fruitfly)/1-143            RYHCQDCGKSYSTYSG--LSK-HQQ-FHCP--------SAEGNQVKKVFSCKNCDKTYVSLGALKMHIRTHTLPCKCPICGKAFSRPWLLQGHIRTHTGEKPFSCQHCNRAFADRSNLRAHMQTHSDVKKYSCPTCT-KSFSRMSLLAKHLQSGCQ
DmESCARGOT_(Fruitfly)/1-143          RYQCPDCQKSYSTFSG--LTK-HQQ-FHCP--------AAEGNQVKKSFSCKDCDKTYVSLGALKMHIRTHTLPCKCNLCGKAFSRPWLLQGHIRTHTGEKPFSCQHCHRAFADRSNLRAHLQTHSDIKKYSCTSCS-KTFSRMSLLTKHSEGGCP
PvSNAIL1_(Limpet)/1-139              RYQCEACQKSYSTFGG--LSK-HRQ-FHC------------SQQVKKEFRCKYCDKSYSSLGALKMHIRTHTLPCKCKLCGKAFSRPWLLQGHIRTHTGEKPFSCQHCGRAFADRSNLRAHLQTHAEIKKYGCKSCS-KTFSRMSLLLKHGESSCM
CgESCARGOT_(Pacific_oyster)/1-139    RYHCDGCTKVYATFSG--LTK-HKQ-FHC------------ASQVKKEFSCKYCDKTYVSLGALKMHIRTHTLPCKCKLCGKAFSRPWLLQGHIRTHTGEKPFRCDHCARAFADRSNLRAHLQTHSDIKKYSCKSCC-KTFSRMSLLLKHEDGCCS
PvSNAIL2_(Limpet)/1-139              RYQCDSCKKSYSTFSG--LSK-HKQ-FHC------------ASQIKKEFNCKYCDKTYVSLGALKMHIRTHTLPCKCKLCGKAFSRPWLLQGHIRTHTGEKPFSCQHCGRAFADRSNLRAHLQTHSDVKKYSCRSCS-KTFSRMSLLLKHEDGCCG
NvSnailA_(Starlet_sea_anemone)/1-139 KHQCHQCNKGYSTPLG--LAK-HQQ-FHC------------NTHRKKSFTCKHCDKIYVSLGALKMHIRTHTLPCKCSICGKAFNRPWLLQGHIRTHTGEKPYQCTNCKRAFADRSNLRAHMQTHAVVKKYSCSRCK-KSFSRMSLLVKHEDSGCP
NvSnailB_(Starlet_sea_anemone)/1-139 KLQCPNCMKGFNALAT--LMR-HQY-FYC-----------PPTQHKRPFHCKYCEKLYDSLGALKMHIRTHTLPCKCKICGKAFSRPWLLQGHVRTHTGEKPYKCTQCQRAFADRSNLRAHLQTHSDVKKYSCKQCS-KSFSRMSLLLKH-EGSCS
AmSNAIL2_(Stony_coral)/1-139         NYECHDCRKSYSSVTS--LLR-HQQ-YHC------------HPNHKRPFHCKYCDKLYVSLGALKMHIRTHTLPCKCKICGKAFSRPWLFQGHIRTHTGEKPYKCPNCQRAFADRSNLRAHLQTHSEVKKYSCGLCS-KSFSRMSLLLKHEASGCP
AdSNAIL2_(Stony_coral)/1-139         NYECHDCRKSYSSVTS--LLR-HQQ-YHC------------HPNHKRPFHCKYCDKLYVSLGALKMHIRTHTLPCKCKICGKAFSRPWLLQGHIRTHTGEKPYKCPNCQRAFADRSNLRAHLQTHSEVKKYSCGLCS-KSFSRMSLLLKHEASGCP
HrSNAIL1_(Leech)/1-136               ------INNRISTNNGNILNNSNKFIDNN------------NISNNNNNSCNYCGRKYNTTGALKMHIRTHTLPCRCDLCGKSFSRPWLLQGHLRTHSGEKPFKCDFCQRAFADKSNLRAHLQTHAQIKRYCCSHCG-KSFSRLPLLLKH-GASCG
TaSNAIL1_(Placazoa)/1-140            QYNTASIPTNITEENI--SVQ-KDDTKDM------------IYHDGKNFVCKHCNKVYASLGALKMHIRTHTLPCKCKICGKAFSRPWLLQGHIRTHTGEKPFGCEKCNRSFADRSNLRAHMQTHAEIKKYSCYKCR-RTFSRWSSLKKHENSSNC
AmSNAIL1_(Stony_coral)/3-147         RNDVDSPGNLNRAKKP--QKR-ERNASTS------DVSQRKNKRTRKEFACKHCDKNYLSLGALKMHIRTHTLPCKCTICGKAFSRPWLLQGHIRTHTGEKPYQCPKCQRAFADRSNLRAHLQTHSSVKKYSCSQCS-RSFSRMSLLVKH-QYSCG
AdSNAIL1_(Stony_coral)/1-134         RAKKPQKRERNASTSD--VSQ--RK----------------NKRTRKEFACKHCDKNYLSLGALKMHIRTHTLPCKCTICGKAFSRPWLLQGHIRTHTGEKPYQCPKCQRAFADRSNLRAHLQTHSSVKKYSCSQCS-RSFSRMSLLVKH-QYSCG
PcSNAIL1_(Jellyfish)/1-139           IVKPSDFIDKSKEQIN--VPK-KSK-VPR------------KGKNSRHYSCKYCDKDYMSLGALKMHIRTHTLPCQCKICGKSFSRPWLLQGHIRTHTGERPFVCSYCGRAFADRSNLRAHMQTHVDVKKYECRKCM-KSFSRMSLLTKHEEFDCT
HmSNAIL1_(Hydra)/1-146               IFNKIENEKLFSTKQI--IIDASRSQNDT-------LSKSKSGKPPRTFSCKYCVKDYMTLGALKMHIRTHTLPCKCPMCGKAFSRPWLLQGHVRTHTGEKPFKCSHCSRAFADRSNQRAHMQTHFESKKLECSKCC-KTFIRFSQLSKHVLIGCD
SpSNAIL_(sea_urchin)/1-139           KYHCPDCGKEYSTFGG--LSK-HRQ-LHC------------DAQNKKTFNCKYCDKEYMSLGALKMHIRTHTLPCKCKFCGKAFSRPWLLQGHIRTHTGEKPFSCPHCQRAFADRSNLRAHLQTHSEVKKYSCKSCG-KTFSRMSLLNKHEESGCI
TsESCARGOT_(Pork_worm)/1-139         RIQCPDCNRSYSTLGG--LSK-HRQ-FHC------------ISQIKRQFGCKFCEKQYSSLGAMKMHIRTHTLPCKCHICGKAFSRPWLLQGHIRTHTGEKPFCCGHCGRAFADRSNLRAHLQTHTHLKKYSCNLCQ-KTFSRMSLLTKHVTSGCR
CiSNAIL_(Ascidia)/1-138              RIPCTECGRTYATIGA--LAK-HAK-THE------------DPESGSKFNCKICKKECSSLGALRMHIRTHTLPCECHICGKAFSRTWLLQGHIRTHTGEKPYQCTVCSRAFADRSNLRAHMQTHETVKRYSCVTCE-KTFSRISLLKRH-QVHCE
HaroSNAIL_(Ascidia)/1-139            RFHCSECGRTYATVGG--LMK-HSK-YHH------------DPENAMTFKCKTCEKEYTSLGALKMHIRTHTLPCKCHICGKAFSRTWLLQGHIRTHTGEKPYQCSVCLRAFADRSNLRAHMQTHQNVKRYACTGCE-KTFSRTSLLNRHRASGCV
SkSNAIL_(Acorn_worms)/1-139          RYQCTECSKSYSTFSG--LTK-HKQ-FHC------------VTQAKKSFSCKYCEKEYLSLGALKMHIRTHTLPCKCKICGKAFSRPWLLQGHIRTHTGEKPFACTYCNRAFADRSNLRAHLQTHSDVKKYCCKSCS-KTFSRMSLLTKHEEAGCC
BfSNAIL_(Amphioxus)/1-139            RYQCPQCAKSYSTYSG--LTK-HKQ-FHC------------VTQSKKAFNCKYCDKVYVSLGALKMHIRTHTLPCKCKICGKAFSRPWLLQGHVRTHTGEKPFACPHCSRAFADRSNLRAHLQTHSDIKKYSCKNCS-KTFSRMSLLTKHEEAGCC
DrSNAIL3_(Zebrafish)/2-140           SDECFDCQKAYLSFSN--LAN-QRQ-VHC------------QWPCHKYFTCKYCEKEYVSLGALKMHIRTHTLPCVCKLCGKAFSRPWLLQGHIRTHTGEKPFTCPHCSRAFADRSNLRAHLQTHSEIKKYQCRNCF-KTFSRISLLTKHEEAGCC
TrSNAIL3_(Pufferfish)/1-139          RFECLDCHNEHLSFMG--LAK-CRQ-LRC------------EWSSKKYFNCKYCEKEYISLGALKMHIRTHTLPCVCKLCGKAFSRPWLLQGHIRTHTGEKPFSCFHCSRAFADRSNLRAHLQTHSEMKRYQCACCL-KTFSRISLLAKHQEAGCP
OoSNAIL3_(Killer_whale)/1-137        RAPCTPGG--FRTPAA--LAR-HQQ-LQC------------HLQAQRCFTCKFCDKEYGSLGALKMHIRTHTLPCLCTVCGKAFSRPWLLQGHIRTHTGEKPYTCPHCSRAFADRSNLRAHLQTHSDTKKYQCKRCA-KTFSRVALLARHEESGCC
HsSNAIL3_(Human)/1-139               GFECFHCHKPYHTLAG--LAR-HRQ-LHC------------HLQVGRVFTCKYCDKEYTSLGALKMHIRTHTLPCTCKICGKAFSRPWLLQGHVRTHTGEKPYACSHCSRAFADRSNLRAHLQTHSDAKKYRCRRCT-KTFSRMSLLARHEESGCC
MmSNAIL3_(Mouse)/1-139               SFECIHCHRPYHTLAG--LAR-HQQ-LHC------------HLPTGRAFTCRYCDKEYASLGALKMHIRTHTLPCICKVCGKAFSRPWLLQGHIRTHTGEKPYTCSHCSRAFADRSNLRAHLQTHVGTKKYRCAVCP-KAFSRMSLLARHEEAGCC
GgSNAIL2_(Chicken)/1-139             KFQCGLCNKTYSTFSG--LAK-HKQ-LHC------------DAQSRKSFSCKYCDKEYVSLGALKMHIRTHTLPCVCKICGKAFSRPWLLQGHIRTHTGEKPFSCPHCNRAFADRSNLRAHLQTHSDVKKYQCKNCS-KTFSRMSLLHKHEESGCC
AcSNAIL2-like_(Anole_lizard)/1-139   KFQCSLCNKTYSTFSG--LAK-HKQ-LHC------------DAQSRKSFSCKYCDKEYVSLGALKMHIRTHTLPCVCKICGKAFSRPWLLQGHIRTHTGEKPFSCPHCNRAFADRSNLRAHLQTHSDVKKYQCKNCS-KTFSRMSLLHKHEESGCC
HsSNAIL2_(Human)/1-139               KFQCNLCNKTYSTFSG--LAK-HKQ-LHC------------DAQSRKSFSCKYCDKEYVSLGALKMHIRTHTLPCVCKICGKAFSRPWLLQGHIRTHTGEKPFSCPHCNRAFADRSNLRAHLQTHSDVKKYQCKNCS-KTFSRMSLLHKHEESGCC
MmSNAIL2_(Mouse)/1-139               KFQCNLCNKTYSTFSG--LAK-HKQ-LHC------------DAQSRKSFSCKYCDKEYVSLGALKMHIRTHTLPCVCKICGKAFSRPWLLQGHIRTHTGEKPFSCPHCNRAFADRSNLRAHLQTHSDVKKYQCKNCS-KTFSRMSLLHKHEESGCC
XlSNAIL2_(African_toad)/1-139        KFQCSLCSKTYSTFSG--LAK-HKQ-LHC------------DAQSRKSFSCKYCEKEYVSLGALKMHIRTHTLPCVCKICGKAFSRPWLLQGHIRTHTGEKPFSCPHCNRAFADRSNLRAHLQTHSDVKKYQCKNCS-KTFSRMSLLHKHEESGCC
TrSLUG_(Pufferfish)/1-139            KFQCSLCSKSYSTYSG--LLK-HKQ-LHC------------DAQARKSFSCKYCEKEYVSLGALKMHIRTHTLPCVCKICGKAFSRPWLLQGHIRTHTGEKPFSCPHCNRAFADRSNLRAHLQTHSDVKKYQCKNCS-KTFSRMSLLHKHEESGCC
DrSLUG_(Zebrafish)/1-139             KFQCGLCNKSYSTYSG--LMK-HKQ-LHC------------DAQSRKSFSCKYCEKEYVSLGALKMHIRTHTLPCVCKMCGKAFSRPWLLQGHIRTHTGEKPFSCPHCSRAFADRSNLRAHLQTHSDVKKYQCKNCS-KTFSRMSLLHKHEESGCC
XlSNAIL1_(African_toad)/1-139        KFQCNLCSKSYSTFAG--LSK-HKQ-LHC------------DSQTRKSFSCKYCEKEYVSLGALKMHIRSHTLPCVCKICGKAFSRPWLLQGHIRTHTGEKPFSCTHCNRAFADRSNLRAHLQTHSDVKKYQCKSCS-RTFSRMSLLHKHEETGCT
StSNAIL1-like_(Western_frog)/1-139   KFQCNQCSKSYSTFAG--LSK-HKQ-LHC------------DSQARKSFSCKYCEKEYVSLGALKMHIRSHTLPCVCKICGKAFSRPWLLQGHIRTHTGEKPFSCTHCNRAFADRSNLRAHLQTHSDVKKYQCKNCS-RTFSRMSLLHKHEETGCT
OaSNAIL1_(Platypus)/1-140            KFSCHQCPKAYATFAG--LSK-HRQLLHC------------DAQPRKSFSCKYCEKEYVSLGALKMHIRSHTLPCVCKMCGKAFSRPWLLQGHIRTHTGEKPFSCTHCNRAFADRSNLRAHLQTHSDVKKYQCKSCS-RTFSRMSLLHKHEETGCS
MgSNAIL1_(Wild_Turkey)/1-139         RFRCAQCAKAYSTFAG--LSK-HKQ-LHC------------DAQTRKSFSCKYCEKEYVSLGALKMHIRSHTLPCVCKMCGKAFSRPWLLQGHIRTHTGEKPFSCTHCNRAFADRSNLRAHLQTHSDVKKYQCKTCS-RTFSRMSLLHKHEETGCS
GgSNAIL1_(Chicken)/1-139             RFRCAQCAKAYSTFAG--LSK-HKQ-LHC------------DAQTRKSFSCKYCEKEYVSLGALKMHIRSHTLPCVCKMCGKAFSRPWLLQGHIRTHTGEKPFSCTHCNRAFADRSNLRAHLQTHSDVKKYQCKTCS-RTFSRMSLLHKHEETGCS
HsSNAIL1_(Human)/3-147               TSVSSLEAEAYAAFPG--LGQVPKQ-LAQ-------LSEAKDLQARKAFNCKYCNKEYLSLGALKMHIRSHTLPCVCGTCGKAFSRPWLLQGHVRTHTGEKPFSCPHCSRAFADRSNLRAHLQTHSDVKKYQCQACA-RTFSRMSLLHKHQESGCS
MmSNAIL1_(Mouse)/3-147               TSASSLEAEAFIAFPG--LGQLPKQ-LAR-------LSVAKDPQSRKIFNCKYCNKEYLSLGALKMHIRSHTLPCVCTTCGKAFSRPWLLQGHVRTHTGEKPFSCSHCNRAFADRSNLRAHLQTHSDVKRYQCQACA-RTFSRMSLLHKHQESGCS
DrSNAIL1-like_(Zebrafish)/1-152      RFQCAHCGKSCSSPAC--LSR-HQL-AHCSPQDGISGRTSSSPAPGPPFHCKHCPKEYNSLGALKMHIRSHTLPCVCSTCGKAFSRPWLLRGHIRTHTGERPFSCPHCNRAFADRSNLRAHLQTHSEVKKYQCGSCSRRTFSRMSLLHKHTLSGCC
TrSNAIL2_(Pufferfish)/1-150          IYHCLHCSNSYSSLSA--LSH-HQL-SHF-PQTQHTSSLPTEVSARPAFHCKHCPKEYTSLGALKMHIRSHTLPCVCPTCGKAFSRPWLLRGHIRTHTGERPFACQLCNRAFADRSNLRAHLQTHSEVKKYQCGSCA-RTFSRMSLLHKHNASGCC
TrSNAIL1_(Pufferfish)/1-140          PRQRMKCTGVMARSSP--PEE-EEEREAP------------ATAARPAFLCKHCPKEYTSLGALKMHIRSHTLPCVCTTCGKAFSRPWLLRGHIRTHTGERPFSCPHCNRAFADRSNLRAHLQTHAEVKKYQCGICS-RTFSRMSLLQKHSSSGCC
StSNAIL1_(Western_frog)/1-140        PQQTSRPRRSNKSRAG--QRE-DKSEAAV------------TAASRPAFFCKHCPKEYNSLGALKMHIRSHTLPCVCPTCGKAFSRPWLLRGHIRTHTGERPFSCPHCNRAFADRSNLRAHLQTHADVKKYQCSTCS-RTFSRMSLLQKHSAAGCC
DrSNAIL1_(Zebrafish)/1-140           PQQTSRPRRSNKSRAG--QRE-DKSEAAV------------TAASRPAFFCKHCPKEYNSLGALKMHIRSHTLPCVCPTCGKAFSRPWLLRGHIRTHTGERPFSCPHCNRAFADRSNLRAHLQTHADVKKYQCSTCS-RTFSRMSLLQKHSAAGCC
```

Dataset 4: SMART predictions of the zinc-finger domain region applied to the sequence file that was used for the phylogenteic tree

gene	domain	start	end	E-value	domain	start	end	E-value	domain	start	end	E-value	domain	start	end	E-value	domain	start	end	E-value
Am SNAIL1_coral					ZnF_C2H2	40	62	3.50E-05	ZnF_C2H2	66	88	1.30E-05	ZnF_C2H2	94	116	2.10E-07	ZnF_C2H2	122	142	0.061
Dr SNAIL1_Zebrafish					ZnF_C2H2	34	56	7.80E-06	ZnF_C2H2	60	82	4.00E-05	ZnF_C2H2	88	110	3.60E-07	ZnF_C2H2	116	136	0.069
St SNAIL1_frog					ZnF_C2H2	34	56	7.80E-06	ZnF_C2H2	60	82	4.00E-05	ZnF_C2H2	88	110	3.60E-07	ZnF_C2H2	116	136	0.069
Tr SNAIL1_Pufferfish					ZnF_C2H2	34	56	1.60E-05	ZnF_C2H2	60	82	0.00011	ZnF_C2H2	88	110	3.60E-07	ZnF_C2H2	116	136	0.079
Hm SNAIL1_Hydra					ZnF_C2H2	40	62	0.00011	ZnF_C2H2	66	88	8.90E-06	ZnF_C2H2	94	116	8.70E-06	ZnF_C2H2	122	142	0.25
Mm SNAIL1_Mouse					ZnF_C2H2	39	61	1.20E-05	ZnF_C2H2	65	87	0.00011	ZnF_C2H2	93	115	1.00E-06	ZnF_C2H2	121	141	0.2
Hs SNAIL1_Human					ZnF_C2H2	39	61	1.20E-05	ZnF_C2H2	65	87	0.00043	ZnF_C2H2	93	115	7.60E-07	ZnF_C2H2	121	141	0.2
Nv SNAILIB_sea_anemone	ZnF_C2H2	2	30	0.25	ZnF_C2H2	34	56	2.20E-05	ZnF_C2H2	60	82	1.80E-05	ZnF_C2H2	88	110	5.00E-07	ZnF_C2H2	116	136	0.11
Ad SNAIL1_coral					ZnF_C2H2	29	51	3.50E-05	ZnF_C2H2	55	77	1.30E-05	ZnF_C2H2	83	105	2.10E-07	ZnF_C2H2	111	131	0.061
Hr SNAIL1_Leech					ZnF_C2H2	31	53	3.30E-05	ZnF_C2H2	57	79	1.90E-05	ZnF_C2H2	85	107	8.80E-07	ZnF_C2H2	113	133	0.36
Tr SNAIL3_Pufferfish					ZnF_C2H2	33	55	1.30E-05	ZnF_C2H2	59	81	4.80E-05	ZnF_C2H2	87	109	1.30E-05	ZnF_C2H2	115	135	0.5
Dr SNAIL3_Zebrafish					ZnF_C2H2	33	55	3.80E-06	ZnF_C2H2	59	81	4.80E-05	ZnF_C2H2	87	109	4.80E-07	ZnF_C2H2	115	135	0.24
Haro SNAIL_Ascidia	ZnF_C2H2	2	24	3.20E-05	ZnF_C2H2	33	55	3.70E-06	ZnF_C2H2	59	81	0.0001	ZnF_C2H2	87	109	5.70E-07	ZnF_C2H2	115	135	0.17
Ci SNAIL_Ascidia	ZnF_C2H2	2	24	8.50E-05	ZnF_C2H2	33	55	3.80E-06	ZnF_C2H2	59	81	0.00016	ZnF_C2H2	87	109	1.10E-07	ZnF_C2H2	115	136	0.024
Oo SNAIL3_Killer_whale					ZnF_C2H2	31	53	1.70E-06	ZnF_C2H2	57	79	0.00018	ZnF_C2H2	85	107	2.70E-07	ZnF_C2H2	113	133	0.15
Mm SNAIL3_Mouse	ZnF_C2H2	2	24	0.0038	ZnF_C2H2	33	55	1.70E-06	ZnF_C2H2	59	81	3.90E-05	ZnF_C2H2	87	109	7.90E-07	ZnF_C2H2	115	135	0.13
Hs SNAIL3_Human	ZnF_C2H2	2	24	0.0093	ZnF_C2H2	33	55	6.80E-07	ZnF_C2H2	59	81	2.70E-05	ZnF_C2H2	87	109	1.60E-06	ZnF_C2H2	115	135	0.16
Ce SCRATCH1_Nematode	ZnF_C2H2	2	24	0.00019	ZnF_C2H2	33	55	0.00012	ZnF_C2H2	59	81	6.40E-06	ZnF_C2H2	87	109	5.90E-07	ZnF_C2H2	115	135	0.064
Sp SCRATCH2-like_Sea_urchir	ZnF_C2H2	2	24	1.70E-06	ZnF_C2H2	33	55	0.00019	ZnF_C2H2	59	81	3.20E-06	ZnF_C2H2	87	109	2.90E-06	ZnF_C2H2	115	135	0.069
Dm SCRATCH1-like_Fruitfly	ZnF_C2H2	2	24	2.60E-05	ZnF_C2H2	33	55	0.00082	ZnF_C2H2	59	81	1.80E-05	ZnF_C2H2	87	109	4.50E-07	ZnF_C2H2	115	135	0.083
Dm SCRATCH2-like_Fruitfly	ZnF_C2H2	2	24	2.10E-06	ZnF_C2H2	31	53	0.00084	ZnF_C2H2	57	79	6.80E-05	ZnF_C2H2	85	107	1.60E-06	ZnF_C2H2	113	133	0.13
Ta SCRATCH_Placazoa	ZnF_C2H2	2	24	1.10E-05	ZnF_C2H2	33	55	0.0003	ZnF_C2H2	59	81	1.10E-06	ZnF_C2H2	87	109	1.00E-06	ZnF_C2H2	115	135	0.086
Bf SCRATCH_Amphioxus	ZnF_C2H2	2	24	2.50E-06	ZnF_C2H2	33	55	0.00031	ZnF_C2H2	59	81	9.40E-07	ZnF_C2H2	87	109	2.90E-06	ZnF_C2H2	115	135	0.071
Ad SCRATCH_Stony_coral	ZnF_C2H2	2	24	1.00E-05	ZnF_C2H2	33	55	0.00023	ZnF_C2H2	59	81	7.30E-07	ZnF_C2H2	87	109	7.50E-08	ZnF_C2H2	115	135	0.14
Nv SCRATCH_sea_anemone	ZnF_C2H2	2	24	8.00E-06	ZnF_C2H2	33	55	0.00047	ZnF_C2H2	59	81	2.50E-06	ZnF_C2H2	87	109	1.30E-07	ZnF_C2H2	115	135	0.066
Dp SCRATCH1_Water_flea	ZnF_C2H2	2	24	1.30E-05	ZnF_C2H2	33	55	0.00039	ZnF_C2H2	59	81	1.30E-06	ZnF_C2H2	87	109	1.60E-06	ZnF_C2H2	115	135	0.22
Mm SCRATCH2_Mouse	ZnF_C2H2	2	24	4.50E-05	ZnF_C2H2	33	55	0.00023	ZnF_C2H2	59	81	2.30E-06	ZnF_C2H2	87	109	2.90E-06	ZnF_C2H2	115	135	0.19
Hs SCRATCH2_Human	ZnF_C2H2	2	24	4.50E-05	ZnF_C2H2	33	55	0.0002	ZnF_C2H2	59	81	2.30E-06	ZnF_C2H2	87	109	2.90E-06	ZnF_C2H2	115	135	0.19
Mm SCRATCH1_Mouse	ZnF_C2H2	2	24	3.40E-05	ZnF_C2H2	33	55	0.00018	ZnF_C2H2	59	81	2.30E-06	ZnF_C2H2	87	109	2.90E-06	ZnF_C2H2	115	135	0.13
Hs SCRATCH1_Human	ZnF_C2H2	2	24	3.40E-05	ZnF_C2H2	33	55	0.00018	ZnF_C2H2	59	81	2.30E-06	ZnF_C2H2	87	109	2.90E-06	ZnF_C2H2	115	135	0.13
Dr SCRATCH2_Zebrafish	ZnF_C2H2	2	24	5.80E-06	ZnF_C2H2	33	55	0.00013	ZnF_C2H2	59	81	6.40E-06	ZnF_C2H2	87	109	6.40E-07	ZnF_C2H2	115	135	0.092
Dr SCRATCH1_Zebrafish	ZnF_C2H2	2	24	5.80E-06	ZnF_C2H2	33	55	0.00022	ZnF_C2H2	59	81	6.10E-07	ZnF_C2H2	87	109	2.90E-06	ZnF_C2H2	115	135	0.1
St SCRATCH1-like_frog	ZnF_C2H2	2	24	8.70E-06	ZnF_C2H2	33	55	0.00022	ZnF_C2H2	59	81	2.00E-06	ZnF_C2H2	87	109	2.90E-06	ZnF_C2H2	115	135	0.09
Gg SCRATCH2_Chicken	ZnF_C2H2	2	24	2.50E-06	ZnF_C2H2	33	55	0.00023	ZnF_C2H2	59	81	7.30E-07	ZnF_C2H2	87	109	5.70E-07	ZnF_C2H2	115	135	0.053
Sp SCRATCH1_Sea_urchin	ZnF_C2H2	2	24	1.20E-05	ZnF_C2H2	33	55	3.20E-05	ZnF_C2H2	59	81	0.00011	ZnF_C2H2	87	109	2.10E-07	ZnF_C2H2	115	135	0.1
Dp SCRATCH-like_Water_flea	ZnF_C2H2	2	24	2.70E-06	ZnF_C2H2	33	55	3.20E-05	ZnF_C2H2	59	81	2.50E-06	ZnF_C2H2	87	109	8.30E-07	ZnF_C2H2	115	135	0.24
Dm SCRATCH2_Fruitfly	ZnF_C2H2	2	24	2.10E-06	ZnF_C2H2	33	55	0.00025	ZnF_C2H2	59	81	3.00E-05	ZnF_C2H2	87	109	2.00E-06	ZnF_C2H2	115	135	0.47
Nv SNAILA_sea_anemone	ZnF_C2H2	2	24	0.00026	ZnF_C2H2	33	55	2.70E-06	ZnF_C2H2	59	81	1.20E-05	ZnF_C2H2	87	109	4.40E-07	ZnF_C2H2	115	135	0.16
Dr SLUG_Zebrafish	ZnF_C2H2	2	24	3.20E-05	ZnF_C2H2	33	55	6.00E-06	ZnF_C2H2	59	81	4.10E-05	ZnF_C2H2	87	109	7.60E-07	ZnF_C2H2	115	135	0.14
Tr SLUG_Pufferfish	ZnF_C2H2	2	24	1.70E-05	ZnF_C2H2	33	55	6.00E-06	ZnF_C2H2	59	81	2.60E-05	ZnF_C2H2	87	109	3.60E-07	ZnF_C2H2	115	135	0.14
Xl SNAIL2_toad	ZnF_C2H2	2	24	2.90E-05	ZnF_C2H2	33	55	6.00E-06	ZnF_C2H2	59	81	2.60E-05	ZnF_C2H2	87	109	3.60E-07	ZnF_C2H2	115	135	0.14
Ac SNAIL2-like_Anole_lizard	ZnF_C2H2	2	24	1.30E-05	ZnF_C2H2	33	55	2.40E-06	ZnF_C2H2	59	81	2.60E-05	ZnF_C2H2	87	109	3.60E-07	ZnF_C2H2	115	135	0.14
Gg SNAIL2_Chicken	ZnF_C2H2	2	24	4.90E-05	ZnF_C2H2	33	55	2.40E-06	ZnF_C2H2	59	81	2.60E-05	ZnF_C2H2	87	109	3.60E-07	ZnF_C2H2	115	135	0.14
Mm SNAIL2_Mouse	ZnF_C2H2	2	24	1.60E-05	ZnF_C2H2	33	55	2.40E-06	ZnF_C2H2	59	81	2.60E-05	ZnF_C2H2	87	109	3.60E-07	ZnF_C2H2	115	135	0.14
Hs SNAIL2_Human	ZnF_C2H2	2	24	1.60E-05	ZnF_C2H2	33	55	2.40E-06	ZnF_C2H2	59	81	2.60E-05	ZnF_C2H2	87	109	3.60E-07	ZnF_C2H2	115	135	0.14
Xl SNAIL1_toad	ZnF_C2H2	2	24	6.30E-05	ZnF_C2H2	33	55	7.50E-06	ZnF_C2H2	59	81	2.60E-05	ZnF_C2H2	87	109	1.20E-06	ZnF_C2H2	115	137	0.2
St SNAIL1-like_frog	ZnF_C2H2	2	24	5.70E-05	ZnF_C2H2	33	55	7.50E-06	ZnF_C2H2	59	81	2.60E-05	ZnF_C2H2	87	109	1.20E-06	ZnF_C2H2	115	137	0.14
Oa Snail1_Platypus	ZnF_C2H2	2	25	0.00076	ZnF_C2H2	34	56	7.50E-06	ZnF_C2H2	60	82	4.10E-05	ZnF_C2H2	88	110	1.20E-06	ZnF_C2H2	116	138	0.2
Gg Snail1_Chicken	ZnF_C2H2	2	24	0.0013	ZnF_C2H2	33	55	7.50E-06	ZnF_C2H2	59	81	4.10E-05	ZnF_C2H2	87	109	1.20E-06	ZnF_C2H2	115	137	0.13
Mg Snail1_Wild_Turkey	ZnF_C2H2	2	24	0.0013	ZnF_C2H2	33	55	7.50E-06	ZnF_C2H2	59	81	4.10E-05	ZnF_C2H2	87	109	1.20E-06	ZnF_C2H2	115	137	0.13
Sp SNAIL_sea_urchin	ZnF_C2H2	2	24	1.20E-05	ZnF_C2H2	33	55	5.40E-06	ZnF_C2H2	59	81	1.90E-05	ZnF_C2H2	87	109	7.10E-07	ZnF_C2H2	115	135	0.096
Ts ESCARGOT_Pork_worm	ZnF_C2H2	2	24	0.00015	ZnF_C2H2	33	55	3.10E-05	ZnF_C2H2	59	81	2.20E-05	ZnF_C2H2	87	109	1.10E-05	ZnF_C2H2	115	135	0.054
Dp ESCARGOT_Water_flea	ZnF_C2H2	2	24	8.40E-05	ZnF_C2H2	36	58	4.90E-06	ZnF_C2H2	62	84	0.00012	ZnF_C2H2	90	112	4.30E-07	ZnF_C2H2	118	138	0.15
Cs SNAIL_Spider	ZnF_C2H2	2	24	3.70E-06	ZnF_C2H2	33	55	8.90E-06	ZnF_C2H2	59	81	2.60E-05	ZnF_C2H2	87	109	7.60E-07	ZnF_C2H2	115	135	0.1
Dm WORNIU_Fruitfly	ZnF_C2H2	2	24	5.30E-05	ZnF_C2H2	37	59	4.60E-06	ZnF_C2H2	63	85	4.60E-06	ZnF_C2H2	91	113	4.80E-07	ZnF_C2H2	119	139	0.044

Pv SNAIL1_Limpet	ZnF_C2H2	2	24	0.00016	ZnF_C2H2	33	55	2.90E-07	ZnF_C2H2	59	81	2.60E-05	ZnF_C2H2	87	109	9.00E-07				
Dm ESCARGOT_Fruitfly	ZnF_C2H2	2	24	1.20E-05	ZnF_C2H2	37	59	2.90E-06	ZnF_C2H2	63	85	2.60E-05	ZnF_C2H2	91	113	9.10E-06	ZnF_C2H2	119	139	0.18
Bf SNAIL_Amphioxus	ZnF_C2H2	2	24	1.90E-05	ZnF_C2H2	33	55	2.10E-06	ZnF_C2H2	59	81	1.80E-05	ZnF_C2H2	87	109	1.00E-06	ZnF_C2H2	115	135	0.12
Sk SNAIL_Acorn_worms	ZnF_C2H2	2	24	4.60E-05	ZnF_C2H2	33	55	1.50E-05	ZnF_C2H2	59	81	1.40E-05	ZnF_C2H2	87	109	1.20E-06	ZnF_C2H2	115	135	0.35
Pv SNAIL2_Limpet	ZnF_C2H2	2	24	0.00077	ZnF_C2H2	33	55	1.80E-06	ZnF_C2H2	59	81	2.60E-05	ZnF_C2H2	87	109	9.00E-07	ZnF_C2H2	115	135	0.25
Cg ESCARGOT_Pacific_oyster	ZnF_C2H2	2	24	0.0023	ZnF_C2H2	33	55	1.10E-06	ZnF_C2H2	59	81	2.60E-05	ZnF_C2H2	87	109	3.30E-06	ZnF_C2H2	115	135	0.48
At SNAIL_spider	ZnF_C2H2	2	24	0.0029	ZnF_C2H2	33	55	5.10E-06	ZnF_C2H2	59	81	2.60E-05	ZnF_C2H2	87	109	1.00E-06	ZnF_C2H2	115	135	0.21
Dm SNAIL_Fruitfly	ZnF_C2H2	2	24	0.00047	ZnF_C2H2	37	59	6.80E-06	ZnF_C2H2	63	85	4.60E-06	ZnF_C2H2	91	113	3.90E-07	ZnF_C2H2	119	139	0.093
Tr SNAIL1-like_Pufferfish	ZnF_C2H2	2	24	0.0075	ZnF_C2H2	44	66	7.00E-06	ZnF_C2H2	70	92	4.00E-05	ZnF_C2H2	98	120	2.90E-06	ZnF_C2H2	126	146	0.56
Dr SNAIL1-like_Zebrafish	ZnF_C2H2	2	24	0.00024	ZnF_C2H2	45	67	6.60E-06	ZnF_C2H2	71	93	9.80E-05	ZnF_C2H2	99	121	3.60E-07				
Ad SNAIL2_coral	ZnF_C2H2	2	24	5.30E-05	ZnF_C2H2	33	55	8.70E-06	ZnF_C2H2	59	81	1.40E-05	ZnF_C2H2	87	109	5.00E-07	ZnF_C2H2	115	135	0.14
Am SNAIL2_Stony_coral	ZnF_C2H2	2	24	5.30E-05	ZnF_C2H2	33	55	8.70E-06	ZnF_C2H2	59	81	2.90E-05	ZnF_C2H2	87	109	5.00E-07	ZnF_C2H2	115	135	0.14
Ta SNAIL1_Placazoa					ZnF_C2H2	34	56	8.30E-07	ZnF_C2H2	60	82	1.40E-05	ZnF_C2H2	88	110	1.30E-06	ZnF_C2H2	116	136	0.14
Pc SNAIL1_Jellyfish					ZnF_C2H2	33	55	4.70E-06	ZnF_C2H2	59	81	7.10E-06	ZnF_C2H2	87	109	9.90E-08	ZnF_C2H2	115	135	0.2

Dataset 4 continued: Sequences used for SMART prediction

```
AmSnail-like Stony cora RNDVDSPGNLNRAKKPQKRERNASTSDVSQRKNKRTRKEFACKHCDKNYLSLGALKMHIRTHTLPCKCTICGKAFSRPWLLQGHIRTHTGEKPYQCPKCQRAFADRSNLRAHLQTHSSVKKYSCSQCSRSFSRMSLLVKHQYSCG
DrSnail1_Zebrafish      PQQTSRPRRSNKSRAGQREDKSEAAVTAASRPAFFCKHCPKEYNSLGALKMHIRSHTLPCVCPTCGKAFSRPWLLRGHIRTHTGERPFSCPHCNRAFADRSNLRAHLQTHADVKKYQCSTCSRTFSRMSLLQKHSAAGCC
StSnail3_frog           PQQTSRPRRSNKSRAGQREDKSEAAVTAASRPAFFCKHCPKEYNSLGALKMHIRSHTLPCVCPTCGKAFSRPWLLRGHIRTHTGERPFSCPHCNRAFADRSNLRAHLQTHADVKKYQCSTCSRTFSRMSLLQKHSAAGCC
TrSnail1_Pufferfish     PRQRMKCTGVMARSSPPEEEEEREAPATAARPAFLCKHCPKEYTSLGALKMHIRSHTLPCVCTTCGKAFSRPWLLRGHIRTHTGERPFSCPHCNRAFADRSNLRAHLQTHAEVKKYQCGICSRTFSRMSLLQKHSSSGCC
HmSnail-like426_Hydra   IFNKIENEKLFSTKQIIIDASRSQNDTLSKSKSGKPPRTFSCKYCVKDYMTLGALKMHIRTHTLPCKCPMCGKAFSRPWLLQGHVRTHTGEKPFKCSHCSRAFADRSNQRAHMQTHFESKKLECSKCCKTFIRFSQLSKHVLIGCD
MmSnail1_Mouse          TSASSLEAEAFIAFPGLGQLPKQLARLSVAKDPQSRKIFNCKYCNKEYLSLGALKMHIRSHTLPCVCTTCGKAFSRPWLLQGHVRTHTGEKPFSCSHCNRAFADRSNLRAHLQTHSDVKRYQCQACARTFSRMSLLHKHQESGCS
HsSnail1_Human          TSVSSLEAEAYAAFPGLGQVPKQLAQLSEAKDLQARKAFNCKYCNKEYLSLGALKMHIRSHTLPCVCGTCGKAFSRPWLLQGHVRTHTGEKPFSCPHCSRAFADRSNLRAHLQTHSDVKKYQCQACARTFSRMSLLHKHQESGCS
NvSnailB_sea_anemone    KLQCPNCMKGFNALATLMRHQYFYCPPTQHKRPFHCKYCEKLYDSLGALKMHIRTHTLPCKCKICGKAFSRPWLLQGHVRTHTGEKPYKCTQCQRAFADRSNLRAHLQTHSDVKKYSCKQCSKSFSRMSLLLKHEGSCS
Ad111963_Stony_coral    RAKKPQKRERNASTSDVSQRKNKRTRKEFACKHCDKNYLSLGALKMHIRTHTLPCKCTICGKAFSRPWLLQGHIRTHTGEKPYQCPKCQRAFADRSNLRAHLQTHSSVKKYSCSQCSRSFSRMSLLVKHQYSCG
HrSnail1_Leech          INNRISTNNGNILNNSNKFIDNNNISNNNNNSCNYCGRKYNTTGALKMHIRTHTLPCRCDLCGKSFSRPWLLQGHLRTHSGEKPFKCDFCQRAFADKSNLRAHLQTHAQIKRYCCSHCGKSFSRLPLLLKHGASCG
TrSnail3_Pufferfish     RFECLDCHNEHLSFMGLAKCRQLRCEWSSKKYFNCKYCEKEYISLGALKMHIRTHTLPCVCKLCGKAFSRPWLLQGHIRTHTGEKPFSCFHCSRAFADRSNLRAHLQTHSEMKRYQCACCLKTFSRISLLAKHQEAGCP
DrSnail3_Zebrafish      SDECFDCQKAYLSFSNLANQRQVHCQWPCHKYFTCKYCEKEYVSLGALKMHIRTHTLPCVCKLCGKAFSRPWLLQGHIRTHTGEKPFTCPHCSRAFADRSNLRAHLQTHSEIKKYQCRNCFKTFSRISLLTKHEEAGCC
HaroSnail_Ascidia       RFHCSECGRTYATVGGLMKHSKYHHDPENAMTFKCKTCEKEYTSLGALKMHIRTHTLPCKCHICGKAFSRTWLLQGHIRTHTGEKPYQCSVCLRAFADRSNLRAHMQTHQNVKRYACTGCEKTFSRTSLLNRHRASGCV
CiSnail_Ascidia         RIPCTECGRTYATIGALAKHAKTHEDPESGSKFNCKICKKECSSLGALRMHIRTHTLPCECHICGKAFSRTWLLQGHIRTHTGEKPYQCTVCSRAFADRSNLRAHMQTHETVKRYSCVTCEKTFSRISLLKRHQVHCE
OoSnail3_Killer_whale   RAPCTPGGFRTPAALARHQQLQCHLQAQRCFTCKFCDKEYGSLGALKMHIRTHTLPCLCTVCGKAFSRPWLLQGHIRTHTGEKPYTCPHCSRAFADRSNLRAHLQTHSDTKKYQCKRCAKTFSRVALLARHEESGCC
MmSnail3_Mouse          SFECIHCHRPYHTLAGLARHQQLHCHLPTGRAFTCRYCDKEYASLGALKMHIRTHTLPCICKVCGKAFSRPWLLQGHIRTHTGEKPYTCSHCSRAFADRSNLRAHLQTHVGTKKYRCAVCPKAFSRMSLLARHEEAGCC
HsSnail3_Human          GFECFHCHKPYHTLAGLARHRQLHCHLQVGRVFTCKYCDKEYTSLGALKMHIRTHTLPCTCKICGKAFSRPWLLQGHVRTHTGEKPYACSHCSRAFADRSNLRAHLQTHSDAKKYRCRRCTKTFSRMSLLARHEESGCC
CeCes1_Nematode         RCVCDKCGKSYATTSNLSRHKQTHRALDSPHAKQCPHCDRVYVSMPALSMHILTHNASHECNVCGKRFSRLWLLQGHLRSHTGLRPFSCAHCGKSFADRSNLRAHMLTHTGDKRFECDKCGRRFALRAYLNRHLETCK
SpScratch2-like Sea urc KYKCNECGKQYATSSNLSRHKQTHRSLDSHLAKKCEVCNKVYVSMPALAMHVLTHNLKHKCNVCHKSFSRPWLLQGHMRSHTGEKPFGCAHCGKAFADRSNLRAHMQTHSAYKNYKCKRCDKSFALKSYLNKHYESACF
DmScratch-like1 Fruitfl CYKCCECGKQYATSSNLSRHKQTHRSLDSQSAKKCNTCGKAYVSMPALAMHLLTHKLSHSCDICGKLFSRPWLLQGHLRSHTGEKPYACVHCGKAFADRSNLRAHMQTHSGDKNFKCHRCNKTFALKSYLNKHLESACL
DmScratch_Fruitfly      KYTCSECGKQYATSSNLSRHKQTHVDSQSAKKCHTCGKAYVSMPALAMHLLTHKLSHSCGVCGKLFSRPWLLQGHLRSHTGEKPYGCAHCGKAFADRSNLRAHMQTHSVDKNFECKRCHKTFALKSYLNKHLESACL
Taznf-like_Placazoa     RYTCAECGKQYATSSNLSRHKQTHRSLDGQLARRCKYCDKAYVSMPALAMHVLTHELAHKCNICGKAFSRSWLLQGHMRSHTGEKPYACATCNKRFADRSNLRAHMQTHSSVKSFQCKNCGKSFALKSYLNKHAESGCC
BfSnail-like_Amphioxus  RYTCNECGKQYATSSNLSRHKQTHRPLDSKLAKTCPTCGKVYVSMPALSMHVLTHQLSHKCDICNKAFSRPWLLQGHMRSHTGEKPFGCAHCGKAFADRSNLRAHMQTHSAFKQYKCKRCNKSFALKSYLNKHYESACF
Adv102984_Stony_coral   RYICAECGKSYATSSNLSRHKQTHRSLDSKLAKRCEHCGKAYVSMPALAMHILTHKLHHKCDVCGKAFSRPWLLQGHMRSHTGERPFMCPECNKAFADRSNLRAHMQTHSPLKQFKCNRCDRTFALKSYLNKHAESACA
NvScratch-like sea anem RYVCAECGKSYATSSNLSRHKQTHRSLDGKLAKRCHQCGKAYVSMPALAMHVLTHKLLHKCEICGKSFSRPWLLQGHKRSHTGERPYSCPECNKAFADRSNLRAHLQTHNPLKQYKCERCDRTFALKSYLNKHLESACS
Dp114328_Water_flea     RYVCGECGKAYATSSNLSRHKQTHRSLESGCARTCPTCGKAYVSMPALSMHLLTHALSHVCPVCSKAFSRPWLLQGHMRSHTGEKPYGCAHCGKAFADRSNLRAHMQTHSATKSHHCGKCHKAFALKSYLNKHLESSCF
MmScratch2_Mouse        RHACAECGKTYATSSNLSRHKQTHRSLDSQLARKCPTCGKAYVSMPALAMHVLTHNLRHKCGVCGKAFSRPWLLQGHMRSHTGEKPFGCAHCGKAFADRSNLRAHMQTHSAFKHYRCRQCDKSFALKSYLHKHCEAACV
HsScratch_Human         RHACAECGKTYATSSNLSRHKQTHRSLDSQLARKCPTCGKAYVSMPALAMHLLTHNLRHKCGVCGKAFSRPWLLQGHMRSHTGEKPFGCAHCGKAFADRSNLRAHMQTHSAFKHYRCRQCDKSFALKSYLHKHCEAACA
MmScratch1_Mouse        RHACGECGKTYATSSNLSRHKQTHRSLDSQLARRCPTCGKVYVSMPAMAMHLLTHDLRHKCGVCGKAFSRPWLLQGHMRSHTGEKPFGCAHCGKAFADRSNLRAHMQTHSAFKHFQCKRCKKSFALKSYLNKHYESACF
HsScratch1_Human        RHACGECGKTYATSSNLSRHKQTHRSLDSQLARRCPTCGKVYVSMPAMAMHLLTHDLRHKCGVCGKAFSRPWLLQGHMRSHTGEKPFGCAHCGKAFADRSNLRAHMQTHSAFKHFQCKRCKKSFALKSYLNKHYESACF
DrScratch2_Zebrafish    RHTCNECGKTYATSSNLSRHKQTHRSLDSKMARKCPTCDKVYVSMPALAMHILTHDLKHKCHVCSKAFSRPWLLQGHMRSHTGEKPFACAHCGKAFADRSNLRAHMQTHSAFKHYSCKRCNKTFALKSYLNKHYESACF
DrScratch1_Zebrafish    RHTCNECGKTYATSSNLSRHKQTHRSLDSKMAKKCPTCGKVYVSMPAMAMHLLTHDLKHKCDICGKAFSRPWLLQGHMRSHTGEKPFGCAHCGKAFADRSNLRAHMQTHSAFKHFKCKRCNKTFVLKSYLNKHYESACF
StScratch1-like_frog    RHSCSECGKSYATSSNLSRHKQTHRSLDSKLAKKCPTCGKVYVSMPAMAMHLLTHDLKHKCETCGKAFSRPWLLQGHMRSHTGEKPFGCAHCGKAFADRSNLRAHMQTHSAFKHYKCKRCSKTFALKSYLNKHYESACF
GgScratch2_Chicken      RHSCPECGKTYATSSNLSRHKQTHRSLDSKMARKCPTCGKAYVSMPALAMHVLTHNLKHKCDVCGKAFSRPWLLQGHMRSHTGEKPFGCSHCGKAFADRSNLRAHMQTHSAFKHYKCKQCEKTFALKSYLNKHYESACF
SpSnail2-like Sea urchi GHSCPECRKSYSTSSNLARHRQTHRSVTDQKARKCPHCEKVYVSMPALSMHIRTHKLGCKCHICGKCFSRPWLLQGHIRTHTGERPFSCPQCGKAFADKSNLRAHVQTHSTIKPYVCQKCNKAFALKSYLYKHVESACY
Dp153927_Water_flea     GHECPDCGKRYSTSSNLARHRQTHRSPADQKARRCPHCDKVYVSVPAFSMHVRTHSQGCKCPYCGKSFSRPWLLQGHIRTHTGEKPFTCQICEKAFADKSNLRAHIQTHSNLKPFTCQRCGKAFALKSYLYKHEESSCM
DmScratch-like2 Fruitfl EHICPECGKKYSTSSNLARHRQTHRSIMDKKARHCPYCEKVYVSMPAYSMHVRTHNQGCECQFCGKRFSRPWLLQGHIRTHTGEKPFKCGVCEKAFADKSNLRAHIQTHSNTKPHTCARCGKAFALKSYLYKHEESSCM
NvSnailA_sea_anemone    KHQCHQCNKGYSTPLGLAKHQQFHCNTHRKKSFTCKHCDKIYVSLGALKMHIRTHTLPCKCSICGKAFNRPWLLQGHIRTHTGEKPYQCTNCKRAFADRSNLRAHMQTHAVVKKYSCSRCKKSFSRMSLLVKHEDSGCP
DrSlug_Zebrafish        KFQCGLCNKSYSTYSGLMKHKQLHCDAQSRKSFSCKYCEKEYVSLGALKMHIRTHTLPCVCKMCGKAFSRPWLLQGHIRTHTGEKPFSCPHCSRAFADRSNLRAHLQTHSDVKKYQCKNCSKTFSRMSLLHKHEESGCC
TrSlug_Pufferfish       KFQCSLCSKSYSTYSGLLKHKQLHCDAQARKSFSCKYCEKEYVSLGALKMHIRTHTLPCVCKICGKAFSRPWLLQGHIRTHTGEKPFSCPHCNRAFADRSNLRAHLQTHSDVKKYQCKNCSKTFSRMSLLHKHEESGCC
XlSnail2_toad           KFQCSLCSKTYSTFSGLAKHKQLHCDAQSRKSFSCKYCEKEYVSLGALKMHIRTHTLPCVCKICGKAFSRPWLLQGHIRTHTGEKPFSCPHCNRAFADRSNLRAHLQTHSDVKKYQCKNCSKTFSRMSLLHKHEESGCC
AcSnail2-like Anole liz KFQCSLCNKTYSTFSGLAKHKQLHCDAQSRKSFSCKYCDKEYVSLGALKMHIRTHTLPCVCKICGKAFSRPWLLQGHIRTHTGEKPFSCPHCNRAFADRSNLRAHLQTHSDVKKYQCKNCSKTFSRMSLLHKHEESGCC
GgSnail2_Chicken        KFQCGLCNKTYSTFSGLAKHKQLHCDAQSRKSFSCKYCDKEYVSLGALKMHIRTHTLPCVCKICGKAFSRPWLLQGHIRTHTGEKPFSCPHCNRAFADRSNLRAHLQTHSDVKKYQCKNCSKTFSRMSLLHKHEESGCC
MmSnail2_Mouse          KFQCNLCNKTYSTFSGLAKHKQLHCDAQSRKSFSCKYCDKEYVSLGALKMHIRTHTLPCVCKICGKAFSRPWLLQGHIRTHTGEKPFSCPHCNRAFADRSNLRAHLQTHSDVKKYQCKNCSKTFSRMSLLHKHEESGCC
HsSnail2_Human          KFQCNLCNKTYSTFSGLAKHKQLHCDAQSRKSFSCKYCDKEYVSLGALKMHIRTHTLPCVCKICGKAFSRPWLLQGHIRTHTGEKPFSCPHCNRAFADRSNLRAHLQTHSDVKKYQCKNCSKTFSRMSLLHKHEESGCC
XlSnail1_toad           KFQCNLCSKSYSTFAGLSKHKQLHCDSQTRKSFSCKYCEKEYVSLGALKMHIRSHTLPCVCKICGKAFSRPWLLQGHIRTHTGEKPFSCTHCNRAFADRSNLRAHLQTHSDVKKYQCKSCSRTFSRMSLLHKHEETGCT
StSnail1_frog           KFQCNQCSKSYSTFAGLSKHKQLHCDSQARKSFSCKYCEKEYVSLGALKMHIRSHTLPCVCKICGKAFSRPWLLQGHIRTHTGEKPFSCTHCNRAFADRSNLRAHLQTHSDVKKYQCKNCSRTFSRMSLLHKHEETGCT
OaSnail-like_Platypus   KFSCHQCPKAYATFAGLSKHRQLLHCDAQPRKSFSCKYCEKEYVSLGALKMHIRSHTLPCVCKMCGKAFSRPWLLQGHIRTHTGEKPFSCTHCNRAFADRSNLRAHLQTHSDVKKYQCKSCSRTFSRMSLLHKHEETGCS
GgSnail1_Chicken        RFRCAQCAKAYSTFAGLSKHKQLHCDAQTRKSFSCKYCEKEYVSLGALKMHIRSHTLPCVCKMCGKAFSRPWLLQGHIRTHTGEKPFSCTHCNRAFADRSNLRAHLQTHSDVKKYQCKTCSRTFSRMSLLHKHEETGCS
MgSnail-like Wild Turke RFRCAQCAKAYSTFAGLSKHKQLHCDAQTRKSFSCKYCEKEYVSLGALKMHIRSHTLPCVCKMCGKAFSRPWLLQGHIRTHTGEKPFSCTHCNRAFADRSNLRAHLQTHSDVKKYQCKTCSRTFSRMSLLHKHEETGCS
LvSnail Green sea urchi KYHCPDCGKEYSTFGGLSKHRQLHCDAQNKKTFNCKYCDKEYMSLGALKMHIRTHTLPCKCKFCGKAFSRPWLLQGHIRTHTGEKPFSCPHCQRAFADRSNLRAHLQTHSEVKKYSCKSCGKTFSRMSLLNKHEESGCI
TsEscargot_Pork_worm    RIQCPDCNRSYSTLGGLSKHRQFHCISQIKRQFGCKFCEKQYSSLGAMKMHIRTHTLPCKCHICGKAFSRPWLLQGHIRTHTGEKPFCCGHCGRAFADRSNLRAHLQTHTHLKKYSCNLCQKTFSRMSLLTKHVTSGCR
DpEscargot_Water_flea   RYQCPDCHKSYSTYCGLTKHQELHCAAQQAANKKSFSCKHCEKVYVSLGALKMHIRTHTLPCKCLLCGKAFSRPWLLQGHIRTHTGEKPFSCPHCSRSFADRSNLRAHLQTHSDVKRYSCKSCGKTFSRMSLLSKHEDGGCG
CsSnail_Spider          RYQCPDCNKSYSTYSGLSKHRLMHCATQAKKSFGCKYCDKVYVSLGALKMHIRTHTLPCKCKLCGKAFSRPWLLQGHIRTHTGEKPFSCPHCSRAFADRSNLRAHLQTHSEVKKYRCKTCSKTFSRMSLLLKHEDGGCA
DmWorniu_Fruitfly       RYHCQDCGKSYSTYSGLSKHQQFHCPSAEGNQVKKVFSCKNCDKTYVSLGALKMHIRTHTLPCKCPICGKAFSRPWLLQGHIRTHTGEKPFSCQHCNRAFADRSNLRAHMQTHSDVKKYSCPTCTKSFSRMSLLAKHLQSGCQ
PvSnail1_Limpet         RYQCEACQKSYSTFGGLSKHRQFHCSQQVKKEFRCKYCDKSYSSLGALKMHIRTHTLPCKCKLCGKAFSRPWLLQGHIRTHTGEKPFSCQHCGRAFADRSNLRAHLQTHAEIKKYGCKSCSKTFSRMSLLLKHGESSCM
DmEscargot_Fruitfly     RYQCPDCQKSYSTFSGLTKHQQFHCPAAEGNQVKKSFSCKDCDKTYVSLGALKMHIRTHTLPCKCNLCGKAFSRPWLLQGHIRTHTGEKPFSCQHCHRAFADRSNLRAHLQTHSDIKKYSCTSCSKTFSRMSLLTKHSEGGCP
BfSnail_Amphioxus       RYQCPQCAKSYSTYSGLTKHKQFHCVTQSKKAFNCKYCDKVYVSLGALKMHIRTHTLPCKCKICGKAFSRPWLLQGHVRTHTGEKPFACPHCSRAFADRSNLRAHLQTHSDIKKYSCKNCSKTFSRMSLLTKHEEAGCC
SkSNail_Acorn_worms     RYQCTECSKSYSTFSGLTKHKQFHCVTQAKKSFSCKYCEKEYLSLGALKMHIRTHTLPCKCKICGKAFSRPWLLQGHIRTHTGEKPFACTYCNRAFADRSNLRAHLQTHSDVKKYCCKSCSKTFSRMSLLTKHEEAGCC
PvSnail2_Limpet         RYQCDSCKKSYSTFSGLSKHKQFHCASQIKKEFNCKYCDKTYVSLGALKMHIRTHTLPCKCKLCGKAFSRPWLLQGHIRTHTGEKPFSCQHCGRAFADRSNLRAHLQTHSDVKKYSCRSCSKTFSRMSLLLKHEDGCCG
CgEscargot Pacific oyst RYHCDGCTKVYATFSGLTKHKQFHCASQVKKEFSCKYCDKTYVSLGALKMHIRTHTLPCKCKLCGKAFSRPWLLQGHIRTHTGEKPFRCDHCARAFADRSNLRAHLQTHSDIKKYSCKSCCKTFSRMSLLLKHEDGCCS
AtSnail_spider          RYFCSACSKNYATYSGLSRHKQFHCITLEKKAFNCKYCDKIYVSLGALKMHIRTHTLPCKCNLCGKAFSRPWLLQGHIRTHTGEKPFACPHCSRAFADRSNLRAHLQTHSDVKKYSCNACSKTFSRMSLLLKHNDGGCG
```

```
DmSnail_Fruitfly        RFKCDECQKMYSTSMGLSKHRQFHCPAAECNQEKKTHSCEECGKLYTTIGALKMHIRTHTLPCKCPICGKAFSRPWLLQGHIRTHTGEKPFQCPDCPRSFADRSNLRAHQQTHVDVKKYACQVCHKSFSRMSLLNKHSSSNCT
TrSnail2_Pufferfish     IYHCLHCSNSYSSLSALSHHQLSHFPQTQHTSSLPTEVSARPAFHCKHCPKEYTSLGALKMHIRSHTLPCVCPTCGKAFSRPWLLRGHIRTHTGERPFACQLCNRAFADRSNLRAHLQTHSEVKKYQCGSCARTFSRMSLLHKHNASGCC
DrSnail2_Zebrafish      RFQCAHCGKSCSSPACLSRHQLAHCSPQDGISGRTSSSPAPGPPFHCKHCPKEYNSLGALKMHIRSHTLPCVCSTCGKAFSRPWLLRGHIRTHTGERPFSCPHCNRAFADRSNLRAHLQTHSEVKKYQCGSCSRRTFSRMSLLHKHTLSGCC
Adv1008519_Stony_coral  NYECHDCRKSYSSVTSLLRHQQYHCHPNHKRPFHCKYCDKLYVSLGALKMHIRTHTLPCKCKICGKAFSRPWLLQGHIRTHTGEKPYKCPNCQRAFADRSNLRAHLQTHSEVKKYSCGLCSKSFSRMSLLLKHEASGCP
AmSnail2_Stony_coral    NYECHDCRKSYSSVTSLLRHQQYHCHPNHKRPFHCKYCDKLYVSLGALKMHIRTHTLPCKCKICGKAFSRPWLFQGHIRTHTGEKPYKCPNCQRAFADRSNLRAHLQTHSEVKKYSCGLCSKSFSRMSLLLKHEASGCP
TaSnail_Placazoa        QYNTASIPTNITEENISVQKDDTKDMIYHDGKNFVCKHCNKVYASLGALKMHIRTHTLPCKCKICGKAFSRPWLLQGHIRTHTGEKPFGCEKCNRSFADRSNLRAHMQTHAEIKKYSCYKCRRTFSRWSSLKKHENSSNC
PcSnail_Jellyfish       IVKPSDFIDKSKEQINVPKKSKVPRKGKNSRHYSCKYCDKDYMSLGALKMHIRTHTLPCQCKICGKSFSRPWLLQGHIRTHTGERPFVCSYCGRAFADRSNLRAHMQTHVDVKKYECRKCMKSFSRMSLLTKHEEFDCT
```
